# Oxford Nanopore Technologies [ONT] Sequencing: Clinical Validation in Genetically Heterogeneous Disorders

**DOI:** 10.3390/genes16111325

**Published:** 2025-11-03

**Authors:** Mario Urtis, Chiara Paganini, Viviana Vilardo, Antonio Tescari, Samantha Minetto, Claudia Cavaliere, Andrea Pilotto, Carmela Giorgianni, Alessia Cattaneo, Marilena Tagliani, Maurizia Grasso, Alexandra Smirnova, Payam Ebadi, Valentina Barzon, Valentina Favalli, Andrea Bimbocci, Marta Baragli, Alberto Magi, Alessandra Renieri, Eloisa Arbustini

**Affiliations:** 1 Centre for Inherited Diseases, Department of Research, Fondazione IRCCS Policlinico San Matteo, 27100 Pavia, Italy; m.urtis@smatteo.pv.it (M.U.); c.paganini@smatteo.pv.it (C.P.); vi.vilardo@smatteo.pv.it (V.V.); a.tescari@smatteo.pv.it (A.T.); samantha.minetto@student.unisi.it (S.M.); cl.cavaliere@smatteo.pv.it (C.C.); a.pilotto@smatteo.pv.it (A.P.); c.giorgianni@smatteo.pv.it (C.G.); a.cattaneo@smatteo.pv.it (A.C.); m.tagliani@smatteo.pv.it (M.T.); m.grasso@smatteo.pv.it (M.G.); a.smirnova@smatteo.pv.it (A.S.); 2 Dipartimento di Medicina Sperimentale, Università del Salento, 73100 Lecce, Italy; 3 Genetica Medica, Azienda Ospedaliero-Universitaria Senese, 53100 Siena, Italy; alessandra.renieri@unisi.it; 4 4bases Italia srl, 27100 Pavia, Italy; payam.ebadi@4bases.it (P.E.); valentina.barzon@4bases.it (V.B.); valentina.favalli@4bases.it (V.F.); 5 Department of Information Engineering, University of Florence, 50139 Florence, Italy; andrea.bimbocci@unifi.it (A.B.); marta.baragli@unifi.it (M.B.); alberto.magi@unifi.it (A.M.)

**Keywords:** short-read sequencing (SRS), long-read sequencing (LRS), Oxford Nanopore Technology (ONT), validation

## Abstract

**Background/Objectives**: Short-read-sequencing (SRS) is currently the standard for genetic testing in inherited human diseases. Intrinsic limitations include PCR dependency, restricted read length, and challenges in identifying structural variants (SVs), copy number variations (CNVs), and intronic small variants (SNVs/indels). Long-read-sequencing (LRS) enables the sequencing of long DNA molecules, detection of deep intronic variants, rapid testing of few samples, and improved resolution of SVs, CNVs, and SNVs/indels. We therefore aimed to validate Oxford Nanopore Technologies (ONT) LRS for potential clinical application. **Methods**: We evaluated the ONT’s ability to detect pathogenic/likely pathogenic (P/LP) variants previously identified by SRS and confirmed via Sanger sequencing, Multiplex-Ligation-dependent-Probe-Amplification (MLPA), or quantitative-PCR (qPCR). In total, 509 samples were analyzed, including 393 with P/LP variants and 116 negative controls. We used CE-IVD panels HEVA pro, CARDIO pro, BRaCA panel, and ClinEX pro (4Bases-CH). Sequencing was performed on MinION, GridION, and PromethION-2 platforms. Data were analyzed using the 4eVAR pipeline. **Results**: ONT successfully identified all P/LP variants across the panels (sensitivity 100%); identified a previously missed CNV in *ENG* gene; precisely defined the breakpoints of a del(13q) (unsuspected and diagnosed as *BRCA2* del ex2–14); improved the coverage profiles in difficult-to-map regions (e.g., ex1 *TGFBR1*, *PSM2CL*); expanded the coverage of out-of-target deep intronic regions; and allowed for the set-up of fast-track tests (<24 h) for urgent clinical needs. **Conclusions**: Our findings demonstrate that ONT LRS provides diagnostic performance comparable to SRS, with significant advantages in resolving complex and previously undetectable variants. Ongoing developments are further increasing read length, expanding detectable targets, and potential clinical applications.

## 1. Introduction

Over the past decade, short-read sequencing (SRS) NGS platforms, typically generating 150–300 base pairs fragments, have become the gold-standard tool for clinical genetic testing. These technologies have made it possible to translate advanced knowledge of molecular bases of genetic diseases into high-impact diagnostic work-up for a broad range of disorders, including malignancies, cardiovascular diseases, neurological conditions, and complex syndromes. Despite the substantial benefits of SRS-based technologies, a significant proportion of genetic disorders remains undiagnosed [1,2,3]. This diagnostic gap is partially attributable to the intrinsic limitations of SRS technologies, whether applied to multigene panels (MGPs), clinical or whole exome sequencing (CES/WES), or whole-genome sequencing (WGS) [4,5,6]. Limitations primarily related to read length include the identification of deep intronic variants (especially in MGPs and exomes), structural variants (SVs), copy number variations (CNVs), short tandem repeats (STRs) [7], and repeat expansion disorders [8]. In addition, SRS approaches frequently encounter challenges in both sequencing and aligning genomic regions that are highly repetitive, GC-rich, or homologous to pseudogenes, resulting in coverage gaps, low mapping quality, and incomplete variant detection [7]. For example, sequencing of PMS2, a gene whose defects are commonly associated with Lynch Syndrome, is notably complicated by the presence of its highly homologous pseudogene *PMS2CL* [9]. Similar challenges are observed for *BRCA1* [10] and *ABCC6*, the latter of which is associated with autosomal recessive pseudoxanthoma elasticum (PXE) when affected by homozygous or compound heterozygous variants [11].

Recently, long-read sequencing (LRS) platforms, such as Pacific Biosciences (PacBio) and Oxford Nanopore Technologies (ONT), introduced the possibility of evaluating long reads in real-time [12] and are candidates for overcoming SRS’s limitations [13,14,15]. LRS technology is increasingly reported in clinical applications, adding advantages such as detection of deep intronic variants, triplet expansions, time-sparing tests, and SV conformations [15,16,17,18,19,20]. In most recent studies, LRS enhanced the detection of pathogenic variants and identified variants previously undetected with other technologies in patients with rare diseases [21]. Long-read DNA and cDNA sequencing identified deep intronic Pathogenic or Likely Pathogenic (P/LP) variants in *BRCA1*, *PALB2*, and *ATM* genes in 8 of the 120 (6%) previously unresolved families; these variants generate intronic pseudoexons that are spliced into transcripts, leading to premature truncations [22]. Previously undetected *DMD* P/LP variants, including single-nucleotide variants (SNVs) and SVs, were identified in 11 unresolved cases [23]. LRS identified homozygous and compound heterozygous mutations in 13 of 34 families with suspected autosomal recessive diseases that had remained undiagnosed by exome sequencing [24]. Therefore, there is a growing interest to validate LRS technology for its potential application as a stand-alone sequencing approach for diagnostic tests. In our institutionally validated diagnostic pipeline, the identification of clinically actionable variants requires confirmatory testing, particularly when genetic findings impact therapeutic decisions or reproductive decisions. SRS-based results are currently verified using orthogonal methods such as Sanger sequencing and Multiplex Ligation-dependent Probe Amplification (MLPA) or quantitative PCR (qPCR) for CNVs.

To explore the translational potential of ONT LRS as a clinical diagnostic tool, we designed a validation protocol within a research project addressing diagnostic innovation (WP6, INNOVA project, code PNC-E3-2022-23683266).

Our study aimed to evaluate the analytical sensitivity, reliability, and potential for the enhanced diagnostic yield of LRS, specifically assessing the performance of ONT in a clinical setting, with the additional goal of establishing it as a complementary tool for quality control within existing diagnostic workflows.

## 2. Materials and Methods

### 2.1. Study Design

**Figure 1:**
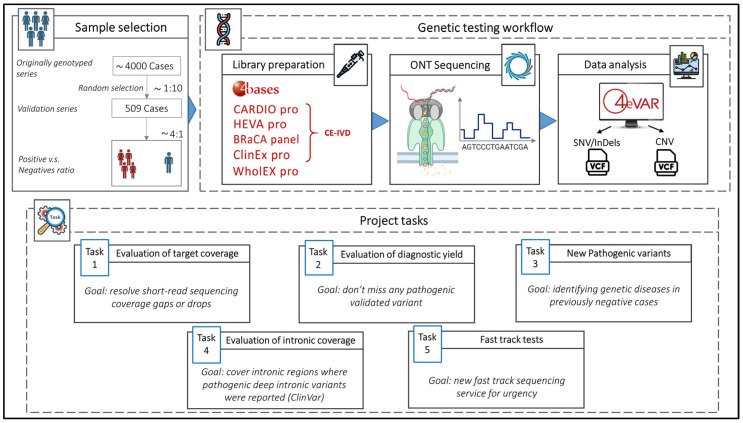
This figure shows the design and workflow of this study from sample selection to data analysis, and the five major tasks of the project.

The design of this study is summarized in Figure 1.

We randomly selected 509 DNA from a series of 4000 residual samples, collected after completion of the diagnostic workflow, previously tested with short read NGS, with MGPs and Clinical Exomes. Each DNA sample was anonymized and labeled with two paired project codes: INNOVA SRS-# for short-read data and INNOVA LRS-# for long read sequencing data. We included both positive (*n* = 393, with one or more LP/P variants) and negative (*n* = 116 with VUS) samples with an approximate 4:1 ratio (Table 1). All SNVs or small insertion/deletions (InDels) previously identified by SRS had been confirmed using Sanger sequencing, while CNVs had been validated using MLPA, qPCR and aCGH, as for institutional diagnostic clinical programs.

### 2.2. Selection of Kits for Library Preparation

We used a CE-IVD amplicon-based BRCA1/2 panel (BRaCA panel) and four different CE-IVD capture-based multigene panels (MGPs) targeting heritable cardiomyopathies and connective tissue diseases (CARDIO pro), solid cancers (HEVA pro), clinical exome (ClinEx pro) and whole exome (WholEX pro) (https://4bases.ch/). The kits (except for WholEX Pro) were selected because they are CE-IVD marked, compatible with Nanopore technology, and the library preparation (target-enrichment) is similar to that currently used for the short read libraries. One sample was sequenced using low-coverage (5×) WGS approach. For sequencing, we used MinION for single/few samples and small panels, GridION for the full batch of multi-gene panels, and PromethION2 (P2) for exome sequencing. The low-coverage WGS experiment was performed on a single GridION flowcell.

### 2.3. Project-Specific Tasks

1. Target coverage. The qualitative evaluation of the target was conducted by inspecting all genes of interest and, in particular, genes with difficult-to-map and difficult-to-capture regions with SRS, such as exons 13–14–15 of PMS2 and exon 1 of TGFBR1. The goal was the full coverage of gaps or drops that affect SRS in key regions for genetic diagnoses.
2. Diagnostic Yield. ONT LRS results were benchmarked with previously detected and validated P/LP variants of our truth set. For each positive sample, the presence of known P/LP variants were assessed. The goal was to achieve a diagnostic yield at least equal to that of gold-standard sequencing systems as required by EU HTA rules for the validation of new health technologies (https://ec.europa.eu/commission/presscorner/detail/en/ip_25_226, accessed on 20 October 2025).
3. Identification of LP/P Variants previously missed using SRS in samples from cases with strong clinical suspicion of genetic disorders.
4. Intronic regions coverage. Intronic regions that are typically inaccessible to SRS targeting exonic regions (MGPs and exonic sequencing) may harbor deep intronic pathogenic variants. For intron coverage, the proportion of the off-target intron sequences was calculated with a minimum sequencing depth of 10× in both ONT LRS and SRS samples. This threshold ensures sufficiently reliable variant calling. Although regions with lower coverage may still allow variant detection, confidence is lower, and the risk of false-negative or uncertain results is higher. In addition, given the possible translational scope of our protocol, a systematic analysis of all gene regions containing known P/LP deep intronic (at least 100 bp-distant from the nearest exon) variants reported in ClinVar was conducted. For each known variant, coverage depth was assessed in both ONT LRS and SRS samples.
5. Fast track sequencing. A fast-track sequencing workflow was developed to tests cases clinically requiring urgent genetic test (rare but possible), where test results can be essential to decision-making tailored to the patient such as pregnancy-associated breast cancer (PABC) [25] or prenatal testing when precedent family genetic data are not available [26]. The goal was to optimize and validate the results, turnaround times, and costs.

All benchmarking analyses comparing the performance of the sequencing technologies were conducted on genes shared between the panels sequenced by ONT LRS and SRS. The complete list of genes included in the benchmarking is provided in Supplementary Table S1.

### 2.4. DNA Isolation

DNA previously extracted from peripheral blood samples by Maxwell RSC Instrument (Promega, Madison, WI, USA) using Maxwell CSC Blood DNA Kit (Promega) according to the manufacturer’s protocol, were used for this study. The DNA concentration was measured by Quantus (Promega) using QuantiFluor ONE dsDNA System kit (Promega), and the DNA quality was checked by NanoDrop 1000 (ThermoFisher Scientific, Waltham, MA, USA).

### 2.5. Libraries Preparation with Capture-Based Technology

For each sample, capture-based library was prepared using the Pro Technologies kit (4bases SA, Manno, Switzerland) according to the manufacturer’s instructions for ONT sequencing. Briefly, the fragmentation by Frag/AT enzyme was performed on 100 ng of DNA, and Universal adapters were added by ligation. After sample purification with DNA Purification Beads, UDI primers were used for indexing. Then, a second purification step was performed and the concentration of DNA was measured by Quantus (Promega) using QuantiFluor ONE dsDNA System kit (Promega). A single library of 1500 ng DNA was created by pooling together barcoded samples. Then, the library was hybridized overnight, using probes targeting specific genes for each panel. After a purification step with Streptavidin Binding Beads, hybridized probe were amplified by PCR protocol. The last purification step was performed to clean up the library and the library’s concentration was determined by Quantus (Promega) using QuantiFluor ONE dsDNA System kit (Promega). Fragment distribution of the final library was assessed using the D5000 ScreenTape Assay on Tape Station 4150 (Agilent Technologies, Santa Clara, CA, USA).

### 2.6. Libraries Preparation with Amplicon-Based Technology

CE-IVD BRaCA panel (4Bases SA, CH) was used to identify variants in *BRCA1* and *BRCA2* genes, according to the manufacturer’s instructions. Briefly, samples of 10 ng of DNA were amplified with primer pools targeting the selected gene regions. After a purification step with DNA Purification Beads, the sample concentration was measured by Quantus (Promega) using QuantiFluor ONE dsDNA System kit (Promega). Then, samples were indexed through a PCR protocol and the amplified samples were cleaned up with DNA Purification Beads. Thus, concentration and fragment length were determined by Quantus (Promega) and Tape Station 4150 (Agilent Technologies), respectively.

### 2.7. Low-Coverage Whole Genome Library Preparation

The DNA sample concentration was determined by Quantus (Promega) using QuantiFluor ONE dsDNA System kit (Promega) and the length of its DNA fragment was measured by Tape Station 4150 (Agilent) using Genomic DNA ScreenTape Assay (Agilent) according the manufacturer’s protocol. Fifty-five fmol of sample were used for whole genome analysis by the Nano Adapter kit (4bases) following the manufacturer’s protocol. The concentration of the eluted library was quantified by Quantus (Promega) using QuantiFluor ONE dsDNA System kit (Promega).

### 2.8. ONT Sequencing

ONT sequencing kit (SQKSLK114, ONT, Oxford, UK) and Nano Adapter (4bases SA, Manno, Switzerland) were used to prepare the libraries (200 fmol DNA) for sequencing, according to the manufacturer’s protocol. The final library was loaded on the Flow Cell R10 version (ONT, Oxford, UK) on the GridION sequencer (ONT). ClinEx pro and WholEX pro libraries were loaded on the Flow Cell R10 version, on the P2 sequencer (ONT). The WGS sample was loaded on a single Flow Cell R10 version (ONT) on the GridION sequencer (ONT). The sequencing lasted 72 h for libraries obtained by capture-based technology (CARDIO pro, HEVA pro, ClinEx pro and WholEX pro) and WGS or two hours for libraries obtained by amplicon-based technology (BRaCA panel). The run was monitored using the MinKNOW v23.07.8 software (ONT).

### 2.9. Base Calling of ONT Reads and Data Analysis

The base calling of raw ONT LRS signal data was performed using Dorado basecall server v7.2.13 with the Super Accurate (SUP) model (for GridION), and Dorado basecall server v7.6.8 (SUP) (for PromethION 2). Demultiplexing was performed using 4bases Demux App v1.1. Reads alignment on the GRCh37 reference genome, variant and CNV calling, and variant annotation were performed using 4eVAR analysis software (v2.0) with CARDIOpro V0, HEVApro V1, BRaCApanel V1, and WESpro V0 pipelines for germline data (https://4evar.4bases.ch/, 4bases SA, Manno, Switzerland).

## 3. Results

### 3.1. Evaluation of Target Coverage

**Figure 2:**
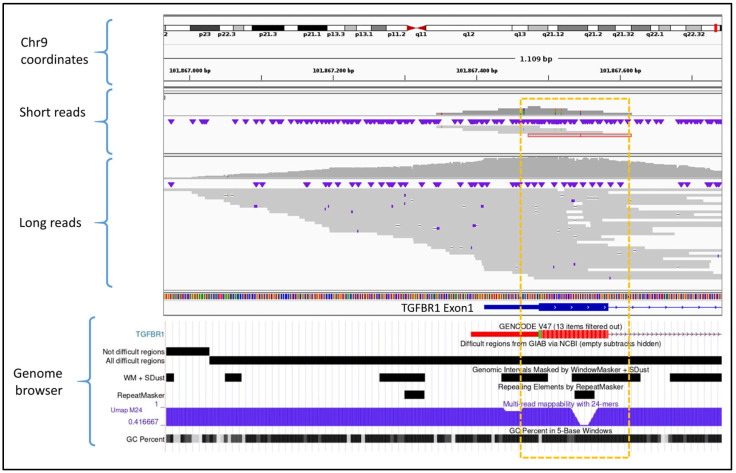
Comparative view of SRS (short reads) versus ONT LRS (long reads) alignments in a difficult-to-map and capture region located in exon 1 of the *TGFBR1* gene. The short-read sample shows limited coverage in the target area (highlighted by the yellow rectangle), with very few reads aligned. In contrast, ONT LRS demonstrates improved coverage due to the increased mappability of long reads in low-complexity or repetitive regions. The genome browser panel at the bottom illustrates the genomic complexity of this region through multiple mapping difficulty scores. The genome track image was obtained from the UCSC Genome Browser (https://genome.ucsc.edu/).

The qualitative assessment of challenging genomic regions revealed that ONT technology significantly improved sequencing performance in gene regions traditionally difficult to map or sequence using SRS platforms. ONT LRS resolved critical coverage issues in specific targets such as exon 1 of the *TGFBR1* gene. This region is characterized by a high GC content and the presence of simple sequence repeats, both of which contribute to its poor performance in SRS (Figure 2). As a result, SRS technologies may fail to produce sufficient coverage for diagnostic interpretation. In contrast, ONT LRS consistently achieved accurate mapping and adequate coverage across this target, thus enabling reliable variant calling even in this difficult genomic region.

**Figure 3:**
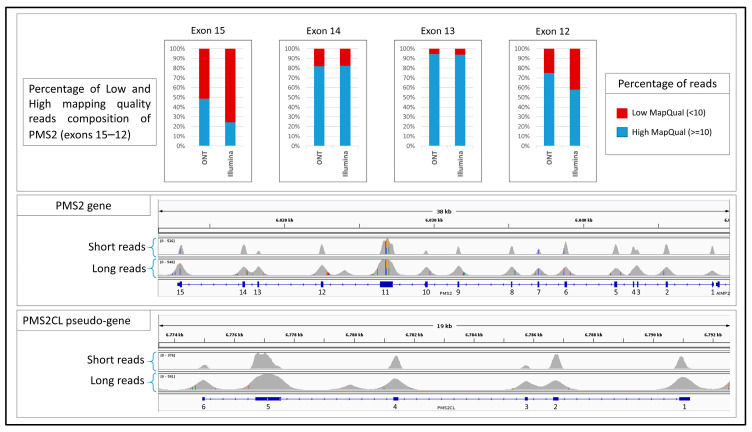
This figure shows the exon-level coverage profiles for the *PMS2* gene, associated with Lynch syndrome, and its highly homologous pseudogene *PMS2CL*. The bar graphs display the proportion of reads with low mapping quality (in red) for both short-read sequencing (SRS) and long-read sequencing (ONT LRS). Notably, exons 12–15 show a substantial difference in mapping quality between the two technologies, reflecting the superior ability of ONT LRS to correctly assign reads in regions of high sequence homology.

ONT LRS demonstrated superior performance in genomic regions characterized by high sequence homology with pseudogenes, such as exons 12 to 15 of the *PMS2* gene. The presence of the highly similar pseudogene *PMS2CL* poses two major challenges. First, during library preparation and target capture, it is technically difficult to enrich selectively *PMS2* exons without co-capturing homologous regions of *PMS2CL*. Second, during read mapping, the high sequence similarity complicates the assignment of the true genomic origin of the sequenced fragments. These issues result in a high proportion of reads with a low mapping quality, leading to a loss of coverage and hampering accurate variant detection. ONT LRS significantly mitigated these issues. LRS consistently improved the coverage of *PMS2* exons 12–15, providing better coverage profiles and reducing the fraction of low-mapping-quality reads compared to SRS (Figure 3). This improvement was pronounced particularly for exons 12 and 15, which are the most affected by pseudogene interference. In these regions, the number of reads with low mapping quality was reduced by approximately 50% with ONT LRS. In the meantime, the correct mapping on *PMS2CL* allowed for the precise recognition of variants present in *PMS2* or *PMS2CL*.

### 3.2. Evaluation of Diagnostic Yield

ONT LRS successfully identified all P/LP variants previously detected by SRS and confirmed by Sanger sequencing or MLPA, demonstrating a diagnostic yield comparable to that of SRS, which is considered the NGS gold standard. With the CARDIO pro panel, a total of 247 variants were identified, including 156 SNVs, 29 InDels, and 31 CNVs. With the HEVA pro panel, 147 variants were identified, including 59 SNVs, 62 InDels, and 26 CNVs. With the BRCA panel, 30 variants were identified, including 15 SNVs, 11 InDels, and 4 CNVs (Table 1; see Supplementary Table S2 and Supplementary Table S3 for the full lists of SNV/InDels and CNVs, respectively). In the validation dataset, 50 samples carried more than one P/LP compound and double variants. Specifically, 36 samples were sequenced with CARDIO pro (two variants in 34 and three variants in 2), and 19 samples with HEVA pro (all had two variants) (see Supplementary Table S4 for the list of double-variants). In all of these samples, ONT LRS successfully detected all second and third variants, ensuring the identification of multiple defects of different genes in the same patient.

### 3.3. Identification of Previously Missed or Incompletely Characterized Pathogenic Variants

**Figure 4:**
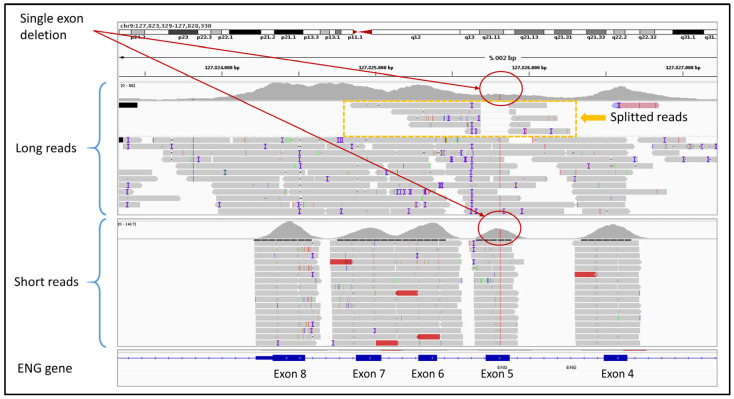
This figure shows the different coverage (red circles) of SRS (short reads) and LRS (long reads) of a previously undetected heterozygous CNV in the exon 5 of the *ENG* gene. LRS clearly shows the exon 5 deletion, also defining precise breakpoints, using split reads (yellow box).

A total of 116 negative samples (VUS and LB/B variants), in which SRS did not identify any P/LP variants, were sequenced using ONT LRS to evaluate whether this approach could detect missed causative variants potentially related to the clinical phenotypes. Among these, 31 samples were sequenced with the CARDIO pro panel, 60 with the HEVA pro panel, and 25 with the BRaCA panel. In none of these cases did ONT identify LP/P variants missed by SRS within the targeted panel genes. An additional set of 12 cases was analyzed with ONT LRS using the ClinEX (*n* = 4) and WholEX pro (*n* = 8) panels. Ten of these had previously tested negative by SRS and MLPA, while in two related individuals a heterozygous deletion affecting exon 5 of the *ENG* gene had been missed by SRS but detected by MLPA. ONT LRS correctly identified the exon 5 deletion in both carriers, which is associated with Osler-Rendu-Weber syndrome 1 (ROS). Upon inspection, it was observed that in SRS data, exon 5 is still captured and partially enriched even in the presence of a deletion, thereby masking the CNV during variant calling. In contrast, ONT sequencing provided a more accurate representation of the true coverage drop caused by the deletion. Furthermore, the presence of split reads spanning the deletion boundaries allowed not only the detection of the CNV but also its precise breakpoint localization (Figure 4).

**Figure 5:**
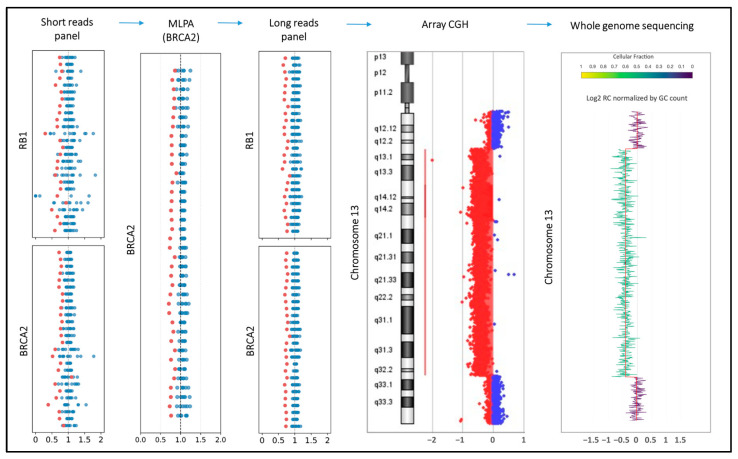
The figure summarizes the findings of multiple genomic technologies targeting chromosome 13. Each dot represents an exon or a probe used to quantify the tested sample (red dots) against control samples (blue dots). The short-read-based panel identified deletions involving both *RB1* and *BRCA2*, with an allelic signal of approximately 0.7 (red) compared to 1.0 in controls (blue), despite an overall low signal quality. MLPA confirmed the complete deletion of *BRCA2* and confirmed the allelic imbalance. Long-read sequencing further validated the deletions of both *RB1* and *BRCA2*, offering higher signal resolution compared to short-read sequencing. Array-CGH identified a deletion spanning the 13q region, confirming the presence of the structural variant and supporting the allelic imbalance. The whole-genome sequencing panel presents the log-transformed read count (RC) profile across chromosome 13, normalized for GC content, as processed using the KaryoSolver tool v.1.0 (4Bases SA, Manno, Switzerland).

In one positive case (SRS-357, Supplementary Table S3), SRS failed to accurately define the extent of the genetic defect, reporting a deletion of *BRCA2* gene and a low quality partial deletion of *RB1* (three noncontiguous exons), which mimicked a false-positive signal. MLPA confirmed a complete deletion of *BRCA2*, but no assay was available for *RB1*. ONT LRS analysis (HEVA pro panel) identified a well-defined deletion in blood cells with an allelic frequency of approximately 30%, encompassing the entire *BRCA2* and *RB1* genes. This result suggested the presence of a larger structural variant, which was confirmed by array CGH as a 13q deletion. To further assess the resolution of ONT LRS in detecting large chromosomal rearrangements, the same sample was tested using low-coverage WGS. Despite the limited depth, ONT LRS WGS analyzed with KaryoSolver (4Bases) successfully confirmed both the presence and allelic imbalance of the structural variant (Figure 5).

### 3.4. Evaluation of Intronic Coverage

**Figure 6:**
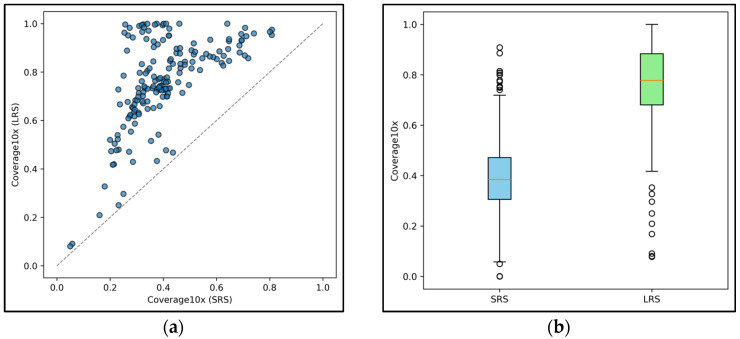
(**a**) The graph shows the percentage of intronic regions covered by ONT LRS and SRS for each gene included in the CARDIO pro and HEVA pro panels. The dashed diagonal line separates the plot into two zones: genes (represented by the blue circles) plotted above the line exhibit higher intronic coverage with ONT LRS compared to SRS, while genes below the line (none in this dataset) indicate better coverage with SRS. (**b**) The graph shows the distribution of aggregated intronic coverage across ONT LRS and SRS samples. White circles represent outliers.

The comparative analysis between LRS and SRS highlights several critical differences in covering intronic regions and in their capacity to detect deep intronic pathogenic variants despite the use of similarly designed capture panels. In a first analysis of 30 samples sequenced with comparable overall mean depth using both ONT LRS and SRS platforms, ONT LRS achieved superior coverage of intronic regions adjacent (flanking) the targeted exons across all genes analyzed. Overall, the intronic region covered by LRS reads was more than doubled compared to that obtained with short-read sequencing (Figure 6). This extended coverage enables variant calling in intronic regions without the need to redesign capture probes beyond the original target.

**Figure 7:**
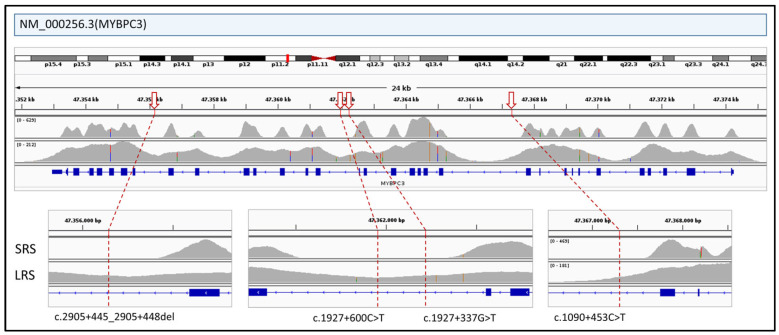
The figure illustrates the differential coverage of intronic regions of the *MYBPC3* gene between SRS and ONT LRS. Several pathogenic deep intronic variants reported in ClinVar (indicated by red arrows; see Supplementary Table S5 for the full list of deep intronic variants) fall within regions that are poorly covered or completely missed by SRS but which are reliably captured by ONT LRS.

Interrogation of ClinVar (https://www.ncbi.nlm.nih.gov/clinvar/ (accessed on 26 April 2025)) identified 44 pathogenic deep intronic variants within genes included in the HEVA pro panel and 50 within the CARDIO pro panel (see Supplementary Table S5 for the full list). In ONT-sequenced samples, 70 of these variants were located in regions with coverage exceeding 10×, which has been defined as the threshold for reliable variant detection. Key example in *MYBPC3* gene is shown in Figure 7. In addition, 10 variants had sub-threshold coverage, and 14 were not covered at all. In contrast, SRS provided sufficient coverage for only 23 variants, with one below the threshold and 70 entirely lacking coverage.

### 3.5. Fast-Track Sequencing

**Figure 8:**
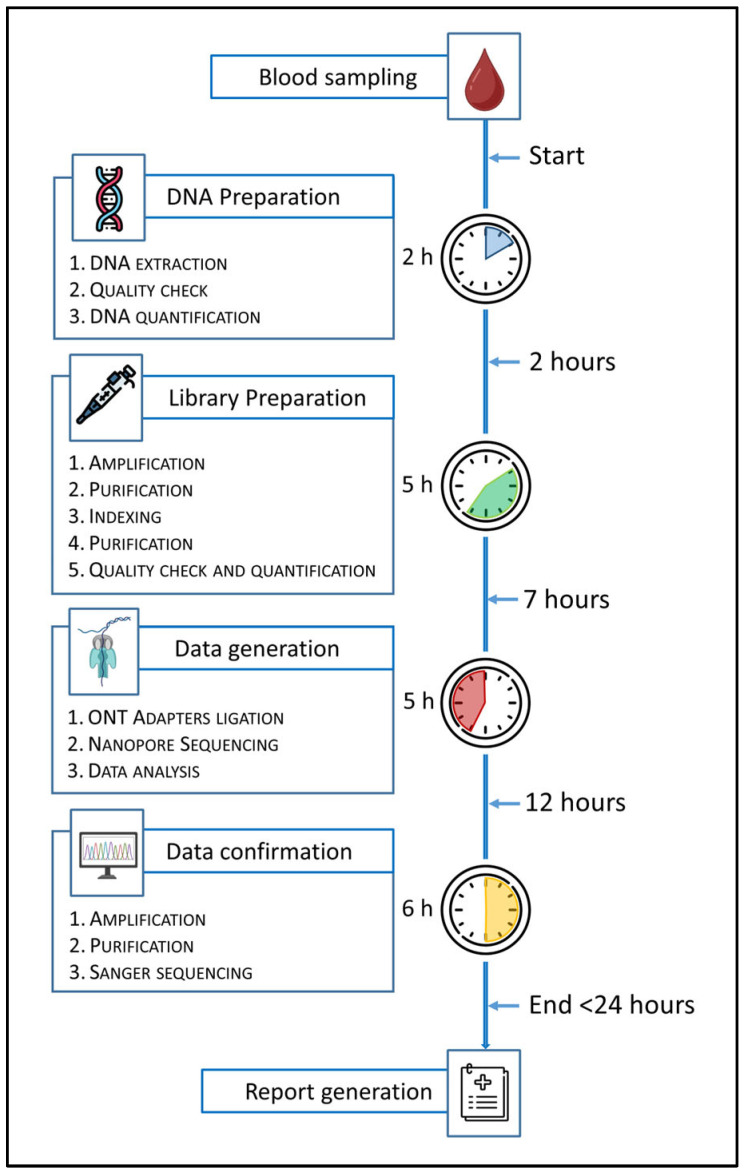
This figure shows the workflow of the fast-track protocol with the specification of the operative steps and their duration from blood sampling to report generation. The fast-track protocol was optimized using the amplicon-based BRaCA panel.

To evaluate the feasibility of a fast diagnostic workflow for urgent clinical scenarios, a fast-track sequencing protocol was implemented on 30 validation samples using the BRaCA panel. This approach was designed to simulate and trial real-world conditions in which a genetic test can be urgently required (e.g., PABC). The workflow consisted of automated DNA extraction, assessment of DNA quality and quantity, amplicon-based library preparation following standard protocols, adapters ligation, sequencing on a MinION or GridION platform, bioinformatic analysis using the 4eVar pipeline (4Bases SA, CH), orthogonal validation of P/LP variants through Sanger sequencing, and finalization of the genetic report (Figure 8). Analysis of turnaround time and associated costs demonstrated that a fast-track workflow based on ONT technology is feasible and well-suited for high-priority clinical cases. While both ONT and SRS technologies offer the same diagnostic efficacy, they differ significantly in operational efficiency, particularly in terms of time-to-result and cost-effectiveness. The time differences are primarily attributed to sequencing runtime. Even with the newer Illumina MiSeq i100 series, the minimum sequencing time is approximately 8 h from loading to run completion. In contrast, ONT platforms offer highly flexible runtimes. In our experiments, optimal diagnostic coverage was achieved within 1 to 2 h of sequencing, resulting in a time saving of at least 6 h. Cost differences arise from the single-use nature of Illumina flow cells versus the reusability of ONT flow cells. Illumina flow cells are consumable per run, which increases the per-sample cost in few-and-small samples scenarios. In contrast, ONT flow cells can be washed and reused for multiple sequencing runs across several days. This feature allows the cost of a single fast-track run to be amortized across a greater number of samples, further improving cost-efficiency. Overall, the ONT-based fast-track workflow emerges as a highly adaptable and economically sustainable solution for urgent genetic testing needs.

## 4. Discussion

As outlined in the study design, the primary objective was to validate ONT LRS for clinical use in alignment with the forthcoming EU Health Technology Assessment (HTA) regulation (2025, http://data.europa.eu/eli/reg/2021/2282/oj (accessed on 20 October 2025)), which evaluates whether any new health technology (Instruments, Devices, Products) performs better, equally well, or worse than existing alternatives. With 100% concordance in the detection of previously confirmed pathogenic or likely pathogenic (P/LP) variants across all CE-IVD marked panels tested in our study, ONT LRS demonstrated diagnostic performance at least equal to short-read sequencing (SRS) technologies currently adopted in clinical practice. Our validation study supports the possible clinical translation of ONT LRS as a reliable and effective approach for genetic diagnostics.

### 4.1. Clinical Implications of ONT Advantages

The advantages of ONT LRS may extend beyond achieving parity in single-nucleotide variant detection. First, ONT LRS demonstrated superior coverage of intronic regions flanking target exons, providing approximately double the genomic coverage compared to conventional short-read approaches. This expanded coverage enables the detection of deep intronic variants that are often missed by standard sequencing methods, thereby offering the potential to increase diagnostic yield in unresolved cases. For instance, approximately 6% of pathogenic variants in *MYBPC3*, a gene associated with hypertrophic cardiomyopathy, are non-canonical splice variants, including deep intronic variants, some of which may be more readily detected through ONT LRS enhanced intronic coverage [27]. A similar challenge exists for the *GLA* gene, implicated in Fabry disease, where a minority of pathogenic variants are deep intronic and population-specific [28]. Moreover, in one of the validated cancer predisposition genes, *APC*, deep intronic germline variants such as c.1408+731C>T (p.Gly471fs), c.1408+735A>T (p.Gly471fs), c.1408+729A>G (p.Gly471fs), and c.532-941G>A (p.Phe178fs) have been predicted to be pathogenic—although these do not appear to be major contributors to colorectal polyposis in the Dutch population [29,30]. Second, ONT LRS significantly improved sequencing performance in challenging genomic regions such as the GC-rich exon 1 of *TGFBR1* and the pseudogene-homologous exons 12–15 of *PMS2*. These loci, which frequently result in coverage gaps or low mapping quality with SRS technologies, were consistently well-covered and accurately mapped by ONT reads. The capacity to resolve such regions without the need for additional enrichment protocols or complex bioinformatic adjustments represents a substantial advancement in clinical sequencing, reducing blind spots in diagnostic gene panels.

Third, ONT LRS demonstrated a clear advantage in the detection and characterization of structural variants, both small and large. Notably, it enabled the identification of a single-exon deletion in the *ENG* gene in a family with Rendu-Osler-Weber disease, which had been missed by SRS due to partial enrichment of the deleted exon, thus masking the CNV during variant calling. Moreover, ONT LRS multi-gene panel testing suggested the presence of a large 13q deletion, which was precisely defined by ONT LRS WGS. This clinically relevant finding was identified in a patient with a history of breast cancer, chronic lymphocytic leukemia, thyroid cancer, and ovarian cystadenomas, and was subsequently confirmed by array-CGH. These examples illustrate ONT’s superior ability to detect CNVs and define their breakpoints, even when located in off-target, intronic, or intergenic regions, where short-read methods may struggle due to intrinsic limitations. This is particularly important in conditions such as dystrophinopathies, where CNVs are a major pathogenic mechanism.

Although the number of additional pathogenic variants detected by ONT LRS in SRS-negative cases was limited, the clinical relevance and diagnostic impact of these findings underscores the complementary role of ONT LRS in selected cases, particularly where structural variants are suspected or where SRS fails to explain a strong clinical phenotype.

These findings provide an excellent example of how LRS, including ONT LRS, could serve as a unique tool with the potential to integrate the diagnostic capabilities of multiple conventional assays (SRS, MLPA, aCGH) into a single comprehensive solution.

### 4.2. Workflow Integration and Implementation

One of the most compelling aspects of ONT lies in its remarkable versatility in turnaround time, which makes it suitable for a wide range of clinical scenarios, from routine diagnostics to urgent settings. Our ONT-based fast-track protocol demonstrated the feasibility of completing the entire workflow within 24 h. This is particularly valuable for time-sensitive clinical scenarios where prompt genetic results can significantly influence clinical decision-making [25]. A growing number of acute clinical conditions or cases requiring fast clinical decisions could benefit from this expanded application of ONT technology in the future.

The economic sustainability of ONT sequencing also deserves consideration. Unlike SRS platforms that rely on single-use flow cells, ONT flow cells can be reused across multiple sequencing runs. This aspect provides a clear cost advantage, especially in low-throughput or urgent single-sample scenarios. This economic efficiency, combined with the technical advantages described above, places ONT as a potentially transformative technology for clinical genetic testing. However, implementation challenges remain. The current research-use-only designation of ONT platforms maintains the need for results orthogonal confirmation via Sanger sequencing or MLPA/qPCR. This dual-method confirmation, required by institutional protocols or scientific guidelines, will remain in place until the ONT instruments and associated workflows are certified for clinical diagnostic use.

### 4.3. Future Directions

In this validation study, CE-IVD multigene panels were primarily employed, as they currently represent the most commonly used approach for the genetic diagnosis of cardiovascular diseases, hereditary cancers, and other rare syndromes investigated at our center. Nevertheless, the progression toward broader genomic analyses using ONT technology from targeted panels to exomes (and potentially WGS) represents a natural evolution that could help address the >50% diagnostic gaps that remain even after conventional exome sequencing. The enhanced ability of ONT to resolve complex genomic regions and structural variants may provide answers in previously unsolved cases, although further validations may be necessary before its widespread clinical adoption.

It is worth noting that this validation focused exclusively on germline variants. Future studies will be essential to evaluate the performance of ONT technology in detecting somatic variants, which are of critical importance in oncology.

A future advantage of ONT technology is its applicability to RNA-based diagnostic studies [31,32,33]. ONT enables the direct sequencing of native RNA molecules, thereby eliminating the need for reverse transcription into cDNA, a process that introduces both analytical bias and additional costs. RNA sequencing for diagnostic purposes could provide direct insights into the functional consequences of genetic variants with unclear effects, such as canonical and non-canonical splicing variants, intronic changes potentially leading to pseudo-exon activation or gene expression modulation via enhancer or silencer regions alteration, and synonymous variants that may activate cryptic exonic splice sites. The possibility of simultaneously sequencing both native DNA and RNA from the same sample foresees a significant advancement in genetic diagnostics, enhancing the interpretation of genomic data and improving the diagnostic yield of testing strategies.

### 4.4. Study Limitations

Two main limitations of our study warrant discussion. First, our approach was primarily designed to validate ONT sequencing versus existing technologies within the framework of gene panels currently used in clinical diagnostic workflows. The panel-based strategy is compatible with read lengths that are substantially shorter than the full capabilities of ONT platforms. In our setting, the obtained reads were approximately 3 to 10 times longer than those generated by short-read sequencing technology, but still far from the ultra-long reads—spanning hundreds of kilobases—that ONT technology can deliver. This choice reflects a deliberate and strategic implementation model, which initially leverages existing laboratory expertise while progressively advancing toward the use of longer reads to fully exploit the capabilities of ONT sequencing. Second, our validation path focused mainly on germline variants in the context of hereditary disorders and cancer predisposition syndromes. Although ONT has demonstrated promising results in various specialized research settings, additional validation will be necessary before extending its application to the routine clinical analysis of somatic alterations.

## 5. Conclusions

Our findings validate ONT sequencing as a reliable and effective tool for clinical genetic testing, with diagnostic yield at least equivalent to current standard-of-care technologies and notable advantages in key clinical areas. The combination of expanded genomic coverage, improved resolution of complex regions, rapid turnaround time, and economic sustainability positions ONT as a valuable addition to the clinical genetics toolkit. The restricted use of ONT for research is a limitation; however, the use of CE-IVD kits/products can overcome it, especially when a second confirmation tool is needed before releasing conclusive diagnostic reports.

## Figures and Tables

**Table 1:** Dataset description.

		HEVA Pro	CARDIO Pro	BRaCA Panel	All Panels
All samples		207	247	55	509
Positive samples (P/LP variants)		147	215	30	393
	SNV	59	155	15	231
	InDels	62	29	11	102
	CNV	26	31	4	61
Negative samples (VUS/LB/B variants)		60	32	25	116

## Data Availability

The original contributions presented in this study are included in the article/Supplementary Material. Further inquiries can be directed to the corresponding author.

## Supplementary Materials

The following supporting information can be downloaded at: https://www.mdpi.com/article/10.3390/genes16111325/s1, Table S1: Genes analyzed for the benchmark; Table S2: Variants detected using the CE-IVD marked multigene panels HEVA pro, Cardio pro, BRaCA (4Bases. CH); Table S3: CNVs; Table S4: Double and compound LP/P variants; Table S5: List of deep intronic variants reported as LP/P in ClinVar within genes included in analyzed panels as detectable with SRS and ONT.

## Abbreviations

The following abbreviations are used in this manuscript:

NGS	Next generation sequencing
SRS	Short-read sequencing
LRS	Long-read sequencing
MGP	Multi-gene panels
CES	Clinical exome sequencing
WES	Whole exome sequencing
WGS	Whole genome sequencing
SV	Structural variants
CNV	Copy number variations
STR	Short tandem repeats
PacBio	Pacific Biosciences
ONT	Oxford Nanopore Technologies
LP/P	Likely pathogenic/pathogenic
MLPA	Multiplex Ligation-dependent Probe Amplification
PCR	Polymerase chain reaction
a-CGH	Array-Comparative Genomic Hybridization
PXE	Pseudoxanthoma elasticum
PABC	Pregnancy-associated breast cancer
ROS	Osler-Rendu-Weber syndrome 1
