# Supplementary material for: Oxford Nanopore Technologies [ONT] Sequencing: Clinical Validation in Genetically Heterogeneous Disorders
Source: Genes (Basel). 2025 Nov 3;16(11):1325. doi: 10.3390/genes16111325 (PMC12652492; doi:10.3390/genes16111325)
Supplement: Supplementary file 1 [file genes-16-01325-s001.zip › genes-3897287-supplementary.pdf]

OXFORD NANOPORE TECHNOLOGIES [ONT] SEQUENCING: CLINICAL VALIDATION IN GENETICALLY HETEROGENEOUS DISORDERS

Mario Urtis[1], Chiara Paganini[1], Viviana Vilardo[1], Antonio Tescari[1,2], Samantha Minetto[1,2,3], Claudia Cavaliere[1], Andrea Pilotto[1], Carmela Giorgianni[1], Alessia Cattaneo[1], Marilena Tagliani[1], Grasso Maurizia[1], Alexandra Smirnova[1], Payam Ebadi[4], Valentina Barzon[4], Valentina Favalli[4], Andrea Bimbocci[5], Marta Baragli[5], Alberto Magi[5], Alessandra Renieri[3], Eloisa Arbustini[1],*

1 Centre for Inherited Diseases, Department of Research, Fondazione IRCCS Policlinico San Matteo, Pavia, Italy;
2 Dipartimento di Medicina Sperimentale, Università del Salento, Lecce, Italy;
3 Genetica Medica, Azienda Ospedaliero-Universitaria Senese, Siena, Italy;
4 4bases Italia srl, Pavia, Italy
5 Department of Information Engineering, University of Florence, Florence, Italy

Corresponding Author
Eloisa Arbustini: e.arbustini@smatteo.pv.it
ORCID: 0000-0003-2948-7994
Phone Number: +39 0382501487
Centre for Inherited Diseases,
Department of Research,
Fondazione IRCCS Policlinico San Matteo,
Piazzale Camillo Golgi 19,
27100, Pavia, Italy

Table S1: Genes analyzed for the benchmark

**CARDIO pro genes**							
ABCC6	ABCC9	ABL1	ACTA1	ACTA2	ACTC1	ACTN2	ADAMTSL4
AKAP9	ALDH18A1	ALPK3	ANK2	ANKRD1	APOA1	B4GALT7	BAG3
BGN	CACNA1C	CACNA2D1	CACNB2	CALM1	CALR3	CASQ2	CAV3
CBS	COL3A1	COL5A1	COL5A2	COL9A1	COL9A3	CRYAB	CSRP3
CTF1	DES	DMD	DOLK	DPP6	DSC2	DSG2	DSP
DTNA	EFEMP2	ELN	EMD	ENG	EYA4	FBN1	FBN2
FHL1	FHL2	FKBP14	FKTN	FLNA	FLNC	FOXE3	GAA
GATA5	GATAD1	GJA1	GJA5	GLA	GPD1L	HCN4	HFE
KCNH2	KCNJ2	KCNJ5	KCNJ8	KCNN1	KCNQ1	LAMA4	LAMP2
LDB3	LMNA	LOX	LTBP2	LTBP3	MAT2A	MFAP5	MYBPC3
MYH11	MYH6	MYH7	MYL2	MYL3	MYLK	MYLK2	MYOZ2
MYPN	NEBL	NEXN	NKX2-5	NOTCH1	NPPA	NUP155	PDLIM3
PKD2	PKP2	PLN	PLOD3	PRDM16	PRKAG2	PRKG1	PSEN1
PSEN2	PTPN11	RAF1	RANGRF	RBM20	ROBO4	RYR2	SCN1B
SCN2B	SCN3B	SCN4B	SCN5A	SCO2	SGCD	SKI	SLC2A10
SLMAP	SMAD2	SMAD3	SMAD4	SMAD6	SNTA1	TAZ	TBX20
TBX5	TCAP	TGFB2	TGFB3	TGFBR1	TGFBR2	TIMP3	TMEM43
TMPO	TNNC1	TNNI3	TNNT2	TNXB	TPM1	TRDN	TRPM4
TTN	TTR	VCAN	VCL				
**HEVA pro genes**							
ALK	APC	ATM	BARD1	BRCA1	BRCA2	BRIP1	CDH1
CDK4	CDKN1C	CDKN2A	CHEK2	EPCAM	FLCN	MEN1	MLH1
MRE11	MSH2	MSH6	MUTYH	NBN	NF1	NF2	PALB2
PMS2	PMS2CL	PTCH1	PTEN	RAD50	RAD51C	RAD51D	RB1
RET	SMAD4	SMARCB1	STK11	TP53	WRN	XPC	XRCC2

Table S2: Variants detected using the CE-IVD marked multigene panels HEVA pro, Cardio pro, BRaCA (4Bases. CH)

SRS-ID	LRS-ID	Gene	HGVSc	HGVSp	Type	Variant type
HEVA pro						
SRS-001	LRS-001	NF1	c.3113+2T>A	-	SNV	Canonical splice variant
SRS-002	LRS-002	APC	c.4761_4762del	p.Ser1588fs	INDEL	Frameshift
SRS-004	LRS-004	PALB2	c.2996+1G>T	-	SNV	Canonical splice variant
SRS-006	LRS-006	BRCA2	c.5851_5854del	p.Ser1951fs	INDEL	Frameshift
SRS-008	LRS-008	CDKN2A	c.301G>T	p.Gly101Trp	SNV	Missense
SRS-011	LRS-011	FLCN	c.1285dup	p.His429fs	INDEL	Frameshift
SRS-013	LRS-013	MLH1	c.1913_1914del	p.Gly638fs	INDEL	Frameshift
SRS-014	LRS-014	MSH2	c.1861C>T	p.Arg621Ter	SNV	Stop gain
SRS-015	LRS-015	MSH6	c.2677_2678del	p.Leu893fs	INDEL	Frameshift
SRS-016	LRS-016	MUTYH	c.1187G>A	p.Gly396Asp	SNV	Missense
SRS-017	LRS-017	NBN	c.994+2T>G	-	SNV	Canonical splice variant
SRS-020	LRS-020	PALB2	c.661_662delinsTA	p.Val221Ter	SNV	Stop gain
SRS-022	LRS-022	PTCH1	c.2577_2583del	p.Phe859fs	INDEL	Frameshift
SRS-023	LRS-023	PTEN	c.1003C>T	p.Arg335Ter	SNV	Stop gain
SRS-024	LRS-024	RAD51C	c.709C>T	p.Arg237Ter	SNV	Stop gain
SRS-025	LRS-025	RAD51D	c.863G>A	p.Trp288Ter	SNV	Stop gain
SRS-027	LRS-027	MUTYH	c.312C>A	p.Tyr104Ter	SNV	Stop gain
SRS-029	LRS-029	TP53	c.523C>T	p.Arg175Cys	SNV	Missense
SRS-262	LRS-262	CHEK2	c.1169A>C	p.Tyr390Ser	SNV	Missense
SRS-263	LRS-263	MSH6	c.3699_3702del	p.Lys1233fs	INDEL	Frameshift
SRS-266	LRS-266	PALB2	c.72del	p.Arg26fs	INDEL	Frameshift
SRS-267	LRS-267	BRCA2	c.4284dup	p.Gln1429fs	INDEL	Frameshift
SRS-268	LRS-268	PMS2	c.2123del	p.Asn708fs	INDEL	Frameshift
SRS-269	LRS-269	MSH2	c.942+3A>G	-	SNV	Non-canonical splice variant
SRS-270	LRS-270	BRCA2	c.658_659del	p.Val220fs	INDEL	Frameshift
SRS-271	LRS-271	MSH2	c.2536C>T	p.Gln846Ter	SNV	Stop gain
SRS-273	LRS-273	BRIP1	c.3731_*5del	c.3731_*5del	INDEL	Start loss
SRS-274	LRS-274	CDKN2A	c.301G>T	p.Gly101Trp	SNV	Missense
SRS-275	LRS-275	CHEK2	c.1427C>T	p.Thr476Met	SNV	Missense
SRS-279	LRS-279	APC	c.3981_3985del	p.Glu1327fs	INDEL	Frameshift
SRS-281	LRS-281	BARD1	c.2050A>T	p.Lys684Ter	SNV	Stop gain
SRS-282	LRS-282	BRCA2	c.5851_5854del	p.Ser1951fs	INDEL	Frameshift
SRS-283	LRS-283	BRCA1	c.190T>C	p.Cys64Arg	SNV	Missense
SRS-284	LRS-284	BRCA2	c.9455_9456del	p.Glu3152fs	INDEL	Frameshift
SRS-285	LRS-285	BRCA1	c.190T>C	p.Cys64Arg	SNV	Missense
SRS-287	LRS-287	MSH2	c.1022_1029del	p.Leu341fs	INDEL	Frameshift
SRS-288	LRS-288	PALB2	c.72del	p.Arg26fs	INDEL	Frameshift
SRS-289	LRS-289	BRCA2	c.2808_2811del	p.Ala938fs	INDEL	Frameshift
SRS-290	LRS-290	BRCA1	c.2722G>T	p.Glu908Ter	SNV	Stop gain
SRS-292	LRS-292	BRCA2	c.2808_2811del	p.Ala938fs	INDEL	Frameshift
SRS-293	LRS-293	BRCA1	c.514del	p.Gln172fs	INDEL	Frameshift
SRS-295	LRS-295	NF1	c.2033dup	p.Ile679fs	INDEL	Frameshift
SRS-296	LRS-296	MUTYH	c.1147del	p.Ala385fs	INDEL	Frameshift
SRS-297	LRS-297	BRCA2	c.6405_6409del	p.Asn2135fs	INDEL	Frameshift
SRS-298	LRS-298	MSH6	c.4024del	p.Arg1342fs	INDEL	Frameshift
SRS-303	LRS-303	CHEK2	c.409C>T	p.Arg137Ter	SNV	Stop gain
SRS-305	LRS-305	BRCA1	c.4964_4982del	p.Ser1655fs	INDEL	Frameshift
SRS-307	LRS-307	MUTYH	c.1437_1439del	p.Glu480del	INDEL	Inframe deletion
SRS-309	LRS-309	PALB2	c.2336C>G	p.Ser779Ter	SNV	Stop gain
SRS-310	LRS-310	CDKN2A	c.259C>T	p.Arg87Trp	SNV	Missense
SRS-312	LRS-312	BRCA2	c.8820_8823del	p.Gln2941fs	INDEL	Frameshift
SRS-314	LRS-314	RAD51C	c.773G>A	p.Arg258His	SNV	Missense
SRS-315	LRS-315	MUTYH	c.536A>G	p.Tyr179Cys	SNV	Missense
SRS-317	LRS-317	BRCA2	c.7976G>A	p.Arg2659Lys	SNV	Missense
SRS-320	LRS-320	MSH2	c.376G>A	p.Gly126Ser	SNV	Missense
SRS-323	LRS-323	BRCA1	c.514del	p.Gln172fs	INDEL	Frameshift

SRS-324	LRS-324	MSH2	c.1901T>G	p.Leu634Ter	SNV	Stop gain
SRS-326	LRS-326	BRCA2	c.7180A>T	p.Arg2394Ter	SNV	Stop gain
SRS-339	LRS-339	FLCN	c.1429C>T	p.Arg477Ter	SNV	Stop gain
SRS-340	LRS-340	MUTYH	c.1147del	p.Ala385fs	INDEL	Frameshift
SRS-341	LRS-341	ATM	c.4906C>T	p.Gln1636Ter	SNV	Stop gain
SRS-350	LRS-350	ATM	c.8484delA	p.Gln2828fs	INDEL	Frameshift
SRS-351	LRS-351	BRCA2	c.8754+2T>A	-	SNV	Canonical splice variant
SRS-352	LRS-352	BRCA2	c.6201del	p.Ile2068fs	INDEL	Frameshift
SRS-353	LRS-353	RAD51C	c.905-2_905-1del	-	INDEL	Canonical splice variant
SRS-354	LRS-354	BRCA1	c.514del	p.Gln172fs	INDEL	Frameshift
SRS-355	LRS-355	PMS2	c.1004A>G	p.Asn335Ser	SNV	Missense
SRS-356	LRS-356	BRCA1	c.3331_3334del	p.Gln1111fs	INDEL	Frameshift
SRS-358	LRS-358	BRCA1	c.815_824dup	p.Thr276fs	INDEL	Frameshift
SRS-359	LRS-359	BRCA1	c.843_846del	p.Ser282fs	INDEL	Frameshift
SRS-360	LRS-360	BRCA1	c.4038_4041del	p.Arg1347fs	INDEL	Frameshift
SRS-361	LRS-361	BRCA2	c.9218A>C	p.Asp3073Ala	SNV	Missense
SRS-364	LRS-364	PMS2	c.1731_1732delinsAGT	p.Arg578fs	INDEL	Frameshift
SRS-365	LRS-365	CHEK2	c.893_897del	p.Tyr298fs	INDEL	Frameshift
SRS-368	LRS-368	ATM	c.8110T>G	p.Cys2704Gly	SNV	Missense
SRS-369	LRS-369	BRCA2	c.9649-2A>G	-	SNV	Canonical splice variant
SRS-370	LRS-370	CDKN2A	c.301G>T	p.Gly101Trp	SNV	Missense
SRS-371	LRS-371	BRCA2	c.3046G>T	p.Glu1016Ter	SNV	Stop gain
SRS-372	LRS-372	RAD50	c.2165dupA	p.Glu723fs	INDEL	Frameshift
SRS-373	LRS-373	PALB2	c.2964delA	p.Val989fs	INDEL	Frameshift
SRS-374	LRS-374	RAD50	c.1739dup	p.Glu584fs	INDEL	Frameshift
SRS-375	LRS-375	PALB2	c.1317delG	p.Phe440fs	INDEL	Frameshift
SRS-376	LRS-376	MSH2	c.1216C>T	p.Arg406Ter	INDEL	Stop gain
SRS-377	LRS-377	BRCA1	c.3464delA	p.Asp1155fs	INDEL	Frameshift
SRS-398	LRS-398	BRCA2	c.5572delA	p.Thr1858fs	INDEL	Frameshift
SRS-404	LRS-404	BARD1	c.513dupA	p.Asp172fs	INDEL	Frameshift
SRS-405	LRS-405	EPCAM	c.712G>T	p.Glu238Ter	SNV	Stop gain
SRS-406	LRS-406	ATM	c.3872T>C	p.Leu1291Pro	SNV	Missense
SRS-407	LRS-407	CHEK2	c.1361G>A	p.Trp454Ter	SNV	Stop gain
SRS-408	LRS-408	ATM	c.7753A>T	p.Lys2585Ter	SNV	Stop gain
SRS-409	LRS-409	BRCA1	c.4032_4034del	p.Asp1344del	INDEL	Inframe deletion
SRS-410	LRS-410	ATM	c.3512A>G	p.Gln1171Arg	SNV	Missense
SRS-411	LRS-411	PMS2	c.1730dupA	p.Arg578fs	INDEL	Frameshift
SRS-412	LRS-412	RAD51C	c.109G>T	p.Glu37Ter	SNV	Stop gain
SRS-413	LRS-413	BRCA2	c.8537_8538del	p.Glu2846fs	INDEL	Frameshift
SRS-446	LRS-446	MUTYH	c.1147delC	p.Ala385fs	INDEL	Frameshift
SRS-447	LRS-447	BRCA2	c.7976G>A	p.Arg2659Lys	SNV	Missense
SRS-448	LRS-448	BRCA2	c.758_762delGTGAA	p.Ser253fs	INDEL	Frameshift
SRS-449	LRS-449	WRN	c.3913C>T	p.Arg1305Ter	SNV	Stop gain
SRS-450	LRS-450	EPCAM	c.712G>T	p.Glu238Ter	SNV	Stop gain
SRS-451	LRS-451	BARD1	c.513dupA	p.Asp172fs	INDEL	Frameshift
SRS-452	LRS-452	ATM	c.2921+1G>A	.	SNV	Stop gain
SRS-453	LRS-453	BRCA2	c.7060C>T	p.Gln2354Ter	SNV	Stop gain
SRS-455	LRS-455	MUTYH	c.536A>G	p.Tyr179Cys	SNV	Missense
SRS-458	LRS-458	BRCA2	c.5796_5797delTA	p.His1932fs	INDEL	Frameshift
SRS-459	LRS-459	BRCA2	c.7954G>A	p.Val2652Met	SNV	Missense
SRS-460	LRS-460	BRCA2	c.470_474delAGTCA	p.Lys157fs	INDEL	Frameshift
SRS-461	LRS-461	BRCA1	c.5171A>G	p.Tyr1724Cys	SNV	Missense
SRS-462	LRS-462	BRCA2	c.3046G>T	p.Glu1016Ter	SNV	Stop gain
SRS-463	LRS-463	EPCAM	c.13C>T	p.Gln5Ter	SNV	Stop gain
SRS-464	LRS-464	TSC2	c.5068+27_5069-47del	.	INDEL	Canonical splice variant
SRS-471	LRS-471	ATM	c.6006+1G>A	.	SNV	Canonical splice variant
SRS-472	LRS-472	MSH6	c.4024del	p.Arg1342fs	INDEL	Frameshift
SRS-474	LRS-474	BRCA2	c.8537_8538del	p.Glu2846fs	INDEL	Frameshift
SRS-475	LRS-475	PMS2	c.1164del	p.His388fs	INDEL	Frameshift
SRS-476	LRS-476	BRCA2	c.7434_7435+17del	.	INDEL	Canonical splice variant
SRS-496	LRS-496	BRCA2	c.8057T>C	p.Leu2686Pro	SNV	Missense
SRS-498	LRS-498	BRCA2	c.10150C>T	p.Arg3384*	SNV	Stop gain

SRS-499	LRS-499	BRCA1	c.4161_4162delTC	p.Gln1388fs	INDEL	Frameshift
SRS-502	LRS-502	MUTYH	c.1147delC	p.Ala385fs	INDEL	Frameshift
SRS-508	LRS-508	WRN	c.1898+2T>G	-	SNV	Canonical splice variant
Cardio pro						
SRS-085	LRS-085	FBN1	c.2737G>A	p.Glu913Lys	SNV	Missense
SRS-087	LRS-087	DMD	c.5632C>T	p.Gln1878Ter	SNV	Stop gain
SRS-088	LRS-088	SMAD3	c.860G>A	p.Arg287Gln	SNV	Missense
SRS-089	LRS-089	KCNQ1	c.1590+1G>A	-	SNV	Canonical splice variant
SRS-090	LRS-090	LMNA	c.590_596del	p.Leu197fs	INDEL	Frameshift
SRS-091	LRS-091	KCNQ1	c.364dup	p.Cys122fs	INDEL	Frameshift
SRS-092	LRS-092	MYBPC3	c.3259A>T	p.Lys1087Ter	SNV	Stop gain
SRS-093	LRS-093	MYH7	c.727C>T	p.Arg243Cys	SNV	Missense
SRS-094	LRS-094	MYL2	c.52T>C	p.Phe18Leu	SNV	Missense
SRS-095	LRS-095	TNNI3	c.575G>A	p.Arg192His	SNV	Missense
SRS-096	LRS-096	TTN	c.103238del	p.His34413fs	INDEL	Frameshift
SRS-097	LRS-097	COL3A1	c.2105G>A	p.Gly702Asp	SNV	Missense
SRS-098	LRS-098	MYH7	c.1988G>A	p.Arg663His	SNV	Missense
SRS-100	LRS-100	TTN	c.32471-1G>A	-	SNV	Canonical splice variant
SRS-101	LRS-101	RYR1	c.4852C>T	p.Arg1618Cys	SNV	Missense
SRS-103	LRS-103	TTN	c.31763-1G>A	-	SNV	Canonical splice variant
SRS-104	LRS-104	MYBPC3	c.2309-2A>G	-	SNV	Canonical splice variant
SRS-105	LRS-105	TTN	c.100897C>T	p.Gln33633Ter	SNV	Stop gain
SRS-106	LRS-106	MYBPC3	c.1624G>C	p.Glu542Gln	SNV	Missense
SRS-108	LRS-108	LAMA2	c.4936G>T	p.Glu1646Ter	SNV	Stop gain
SRS-109	LRS-109	FBN1	c.3337+1G>A	-	SNV	Canonical splice variant
SRS-110	LRS-110	PKP2	c.2578-1G>A	-	SNV	Canonical splice variant
SRS-112	LRS-112	MYH7	c.2572C>T	p.Arg858Cys	SNV	Missense
SRS-113	LRS-113	MYBPC3	c.373_374del	p.Ala125Ter	INDEL	Stop gain
SRS-114	LRS-114	LMNA	c.568C>T	p.Arg190Trp	SNV	Missense
SRS-115	LRS-115	FLNC	c.19_26dup	p.Ala10fs	INDEL	Frameshift
SRS-116	LRS-116	MYBPC3	c.2737+2_2737+3del	-	INDEL	Canonical splice variant
SRS-118	LRS-118	TTN	c.104069del	p.Gly34690fs	INDEL	Frameshift
SRS-120	LRS-120	RYR1	c.14903C>T	p.Pro4968Leu	SNV	Missense
SRS-122	LRS-122	MYH7	c.4900C>T	p.Arg1634Cys	SNV	Missense
SRS-123	LRS-123	ACTA2	c.169G>A	p.Gly57Ser	SNV	Missense
SRS-124	LRS-124	FBN1	c.2668T>A	p.Cys890Ser	SNV	Missense
SRS-125	LRS-125	TTN	c.1245+2T>C	-	SNV	Canonical splice variant
SRS-127	LRS-127	DSP	c.8039_8042del	p.Ile2680fs	INDEL	Frameshift
SRS-130	LRS-130	FBN1	c.4511A>G	p.Asn1504Ser	SNV	Missense
SRS-131	LRS-131	FBN1	c.5162G>A	p.Cys1721Tyr	SNV	Missense
SRS-134	LRS-134	MYBPC3	c.3713T>C	p.Leu1238Pro	SNV	Missense
SRS-135	LRS-135	MT-TL1	m.3243A>G	-	SNV	Mitochondrial
SRS-136	LRS-136	TRPM4	c.1249del	p.Asp417fs	INDEL	Frameshift
SRS-139	LRS-139	FLNC	c.7233_7236del	p.Thr2412fs	INDEL	Frameshift
SRS-140	LRS-140	MYH7	c.5287G>A	p.Ala1763Thr	SNV	Missense
SRS-143	LRS-143	TNNI3	c.484C>T	p.Arg162Trp	SNV	Missense
SRS-144	LRS-144	MYH7	c.1451A>C	p.Lys484Thr	SNV	Missense
SRS-145	LRS-145	NEBL	c.267C>G	p.Tyr89Ter	SNV	Stop gain
SRS-146	LRS-146	SCN5A	c.4719C>T	p.Gly1573Gly	SNV	Synonimus
SRS-147	LRS-147	PTPN11	c.1472C>A	p.Pro491His	SNV	Missense
SRS-148	LRS-148	LMNA	c.1634G>A	p.Arg545His	SNV	Missense
SRS-149	LRS-149	TNNT2	c.833G>C	p.Arg278Pro	SNV	Missense
SRS-150	LRS-150	MYH7	c.728G>A	p.Arg243His	SNV	Missense
SRS-151	LRS-151	DSP	c.1067C>T	p.Thr356Met	SNV	Missense
SRS-152	LRS-152	TNNI3	c.204del	p.Arg69fs	INDEL	Frameshift
SRS-153	LRS-153	KCNQ1	c.1189C>T	p.Arg397Trp	SNV	Missense
SRS-156	LRS-156	CBS	c.253G>A	p.Gly85Arg	SNV	Missense
SRS-158	LRS-158	LMNA	c.1510G>T	p.Ala504Ser	SNV	Missense
SRS-159	LRS-159	LMNA	c.565C>T	p.Arg189Trp	SNV	Missense
SRS-160	LRS-160	MYH7	c.4076G>A	p.Arg1359His	SNV	Missense
SRS-161	LRS-161	MYBPC3	c.1828G>C	p.Asp610His	SNV	Missense
SRS-162	LRS-162	MYBPC3	c.772G>A	p.Glu258Lys	SNV	Missense

SRS-164	LRS-164	GLA	c.547+3A>G	-	SNV	Non-canonical splice variant
SRS-165	LRS-165	GLA	c.547+3A>G	-	SNV	Non-canonical splice variant
SRS-167	LRS-167	FBN1	c.299G>T	p.Cys100Phe	SNV	Missense
SRS-168	LRS-168	FBN1	c.299G>T	p.Cys100Phe	SNV	Missense
SRS-171	LRS-171	DMD	c.6283C>T	p.Arg2095Ter	SNV	Stop gain
SRS-172	LRS-172	KCNQ1	c.502G>A	p.Gly168Arg	SNV	Missense
SRS-173	LRS-173	LMNA	c.656A>C	p.Lys219Thr	SNV	Missense
SRS-174	LRS-174	LMNA	c.466C>T	p.Arg156Cys	SNV	Missense
SRS-175	LRS-175	PKP2	c.2119C>T	p.Gln707Ter	SNV	Stop gain
SRS-176	LRS-176	MYH7	c.2167C>A	p.Arg723Ser	SNV	Missense
SRS-177	LRS-177	COL3A1	c.1347+1G>A	-	SNV	Canonical splice variant
SRS-178	LRS-178	RYR1	c.13225_13226del	p.Asp4409fs	INDEL	Frameshift
SRS-179	LRS-179	ACTA2	c.536G>A	p.Arg179His	SNV	Missense
SRS-182	LRS-182	MYH7	c.2631G>T	p.Met877Ile	SNV	Missense
SRS-183	LRS-183	MYBPC3	c.3192dup	p.Lys1065fs	INDEL	Frameshift
SRS-184	LRS-184	MYH7	c.4286T>C	p.Met1429Thr	SNV	Missense
SRS-185	LRS-185	FBN1	c.2293+5G>A	-	SNV	Non-canonical splice variant
SRS-186	LRS-186	MYBPC3	c.2309-1G>C	-	SNV	Canonical splice variant
SRS-187	LRS-187	MYBPC3	c.1724del	p.Gly575fs	INDEL	Frameshift
SRS-189	LRS-189	LMNA	c.949G>A	p.Glu317Lys	SNV	Missense
SRS-190	LRS-190	FBN1	c.7783G>A	p.Gly2595Ser	SNV	Missense
SRS-191	LRS-191	PKP2	c.1841T>C	p.Leu614Pro	SNV	Missense
SRS-192	LRS-192	SCN5A	c.3673G>A	p.Glu1225Lys	SNV	Missense
SRS-193	LRS-193	MYBPC3	c.3G>C	p.Met1?	SNV	Start loss
SRS-194	LRS-194	MT-TL1	m.3243A>G	-	SNV	Mitochondrial
SRS-195	LRS-195	MT-TL1	m.3243A>G	-	SNV	Mitochondrial
SRS-197	LRS-197	TGFBR2	c.1346A>G	p.Tyr449Cys	SNV	Missense
SRS-198	LRS-198	MYH7	c.5404C>A	p.Gln1802Lys	SNV	Missense
SRS-199	LRS-199	DES	c.634C>T	p.Arg212Ter	SNV	Stop gain
SRS-200	LRS-200	MYBPC3	c.3331-1G>A	-	SNV	Canonical splice variant
SRS-201	LRS-201	MYBPC3	c.3732C>A	p.Cys1244Ter	SNV	Stop gain
SRS-202	LRS-202	MYH7	c.1208G>A	p.Arg403Gln	SNV	Missense
SRS-203	LRS-203	MYH7	c.2167C>T	p.Arg723Cys	SNV	Missense
SRS-206	LRS-206	MT-TL1	m.3243A>G	-	SNV	Mitochondrial
SRS-207	LRS-207	MT-TL1	m.3243A>G	-	SNV	Mitochondrial
SRS-208	LRS-208	LMNA	c.1634G>A	p.Arg545His	SNV	Missense
SRS-210	LRS-210	TGFBR1	c.1460G>A	p.Arg487Gln	SNV	Missense
SRS-214	LRS-214	LMNA	c.1039G>A	p.Glu347Lys	SNV	Missense
SRS-215	LRS-215	MYBPC3	c.2182G>T	p.Glu728Ter	SNV	Stop gain
SRS-216	LRS-216	GAA	c.784G>A + c.2238G>C	p.Trp746Cys	SNV	Missense
SRS-217	LRS-217	FBN1	c.6872-14A>G	-	SNV	Non-canonical splice variant
SRS-219	LRS-219	TGFB2	c.631C>T	p.Arg211Cys	SNV	Missense
SRS-220	LRS-220	TGFB3	c.884dup	p.Gln296fs	INDEL	Frameshift
SRS-222	LRS-222	LAMA4	c.5206+1G>A	-	SNV	Canonical splice variant
SRS-223	LRS-223	FBN1	c.7754T>C	p.Ile2585Thr	SNV	Missense
SRS-224	LRS-224	FBN1	c.6799A>C	p.Asn2267His	SNV	Missense
SRS-227	LRS-227	MYH7	c.2134C>T	p.Arg712Cys	SNV	Missense
SRS-228	LRS-228	TGFBR1	c.1052A>G	p.Asp351Gly	SNV	Missense
SRS-231	LRS-231	SCN5A	c.3157G>A	p.Glu1053Lys	SNV	Missense
SRS-232	LRS-232	MYBPC3	c.2429G>A	p.Arg810His	SNV	Missense
SRS-234	LRS-234	FBN1	c.4567C>T	p.Arg1523Ter	SNV	Stop gain
SRS-235	LRS-235	FBN1	c.5268_5280del	p.Ile1757fs	INDEL	Frameshift
SRS-237	LRS-237	LMNA	c.861_864del	p.Ala288fs	INDEL	Frameshift
SRS-238	LRS-238	PKP2	c.2013del	p.Lys672fs	INDEL	Frameshift
SRS-240	LRS-240	FBN1	c.6662G>A	p.Cys2221Tyr	SNV	Missense
SRS-241	LRS-241	MYH7	c.2287G>A	p.Val763Met	SNV	Missense
SRS-243	LRS-243	FBN1	c.6354C>T	p.Ile2118Ile	SNV	Synonimus
SRS-244	LRS-244	MYBPC3	c.98_99del	p.Thr33fs	INDEL	Frameshift

SRS-245	LRS-245	MYH6	c.2326_2329dup	p.Glu777fs	INDEL	Frameshift
SRS-246	LRS-246	SCN5A	c.1061G>T	p.Ser354Ile	SNV	Missense
SRS-248	LRS-248	RYR2	c.497C>G	p.Ser166Cys	SNV	Missense
SRS-249	LRS-249	LMNA	c.579del	p.Glu194fs	INDEL	Frameshift
SRS-250	LRS-250	TGFBR1	c.1022T>C	p.Val341Ala	SNV	Missense
SRS-251	LRS-251	FLNA	c.7157-1G>C	-	SNV	Canonical splice variant
SRS-253	LRS-253	FBN1	c.3146G>C	p.Gly1049Ala	SNV	Missense
SRS-254	LRS-254	SMAD3	c.221G>A	p.Arg74Gln	SNV	Missense
SRS-255	LRS-255	FBN1	c.6087C>G	p.Cys2029Trp	SNV	Missense
SRS-257	LRS-257	MYBPC3	c.406+1G>A	-	SNV	Canonical splice variant
SRS-258	LRS-258	FBN1	c.299G>T	p.Cys100Phe	SNV	Missense
SRS-259	LRS-259	MYH7	c.2174T>G	p.Leu725Arg	SNV	Missense
SRS-342	LRS-342	MYBPC3	c.292+1G>A	-	SNV	Canonical splice variant
SRS-343	LRS-343	SMAD3	c.1102C>T	p.Arg368Ter	SNV	Stop gain
SRS-344	LRS-344	LMNA	c.1004G>A	p.Arg335Gln	SNV	Missense
SRS-345	LRS-345	FLNC	c.4533del	p.Tyr1513fs	INDEL	Frameshift
SRS-348	LRS-348	FBN1	c.5596A>G	p.Ile1866Val	SNV	Missense
SRS-349	LRS-349	RYR1	c.13225_13226del	p.Asp4409fs	INDEL	Frameshift
SRS-382	LRS-382	MYH7	c.2167C>A	p.Arg723Ser	SNV	Missense
SRS-383	LRS-383	TTN	c.42527_42565delinsTCCC	p.Lys14176fs	INDEL	Frameshift
SRS-384	LRS-384	LMNA	c.481G>A	p.Glu161Lys	SNV	Missense
SRS-386	LRS-386	KCNQ1	c.1032G>A	p.Ala344Ala	SNV	Missense
SRS-389	LRS-389	DES	c.1048C>T	p.Arg350Trp	SNV	Missense
SRS-390	LRS-390	TTN	c.72945C>A	p.Tyr24315Ter	SNV	Stop gain
SRS-391	LRS-391	MYH7	c.1207C>T	p.Arg403Trp	SNV	Missense
SRS-392	LRS-392	COL3A1	c.547G>A	p.Gly183Ser	SNV	Missense
SRS-393	LRS-393	TTN	c.70162C>T	p.Arg23388Ter	SNV	Stop gain
SRS-395	LRS-395	TNNI3	c.607C>T	p.Arg203Cys	SNV	Missense
SRS-397	LRS-397	TTN	c.70394C>G	p.Ser23465Ter	SNV	Stop gain
SRS-414	LRS-414	FBN1	c.4210+1del	-	INDEL	Canonical splice variant
SRS-415	LRS-415	SMAD3	c.1102C>T	p.Arg368Ter	SNV	Stop gain
SRS-416	LRS-416	FBN1	c.1949G>C	p.Arg650Pro	SNV	Missense
SRS-417	LRS-417	MYBPC3	c.2905+1G>A	-	SNV	Canonical splice variant
SRS-419	LRS-419	MYBPC3	c.772G>A	p.Glu258Lys	SNV	Missense
SRS-420	LRS-420	MYH7	c.2491A>C	p.Lys831Gln	SNV	Missense
SRS-421	LRS-421	FBN1	c.4747+1G>T	-	SNV	Canonical splice variant
SRS-422	LRS-422	TTN	c.69412+1G>C	-	SNV	Canonical splice variant
SRS-423	LRS-423	TNNT2	c.305G>A	p.Arg102Gln	SNV	Missense
SRS-424	LRS-424	LMNA	c.1634G>A	p.Arg545His	SNV	Missense
SRS-425	LRS-425	TNNI3	c.430C>T	p.Arg144Trp	SNV	Missense
SRS-426	LRS-426	ACTA2	c.403T>C	p.Tyr135His	SNV	Missense
SRS-427	LRS-427	FBN1	c.8080C>T	p.Arg2694Ter	SNV	Stop gain
SRS-428	LRS-428	SMAD3	c.788C>A	p.Pro263His	SNV	Missense
SRS-429	LRS-429	TTN	c.96838C>T	p.Gln32280Ter	SNV	Stop gain
SRS-479	LRS-479	MYBPC3	c.1224-52G>A	.	SNV	Intronic
SRS-480	LRS-480	PTPN11	c.188A>G	p.Tyr63Cys	SNV	Missense
SRS-481	LRS-481	MYBPC3	c.1624G>C	p.Glu542Gln	SNV	Missense
SRS-482	LRS-482	TTN	c.86799_86802del	p.Gly28936Ter	SNV	Stop gain
SRS-483	LRS-483	TTN	c.88765G>T	p.Glu29589Ter	SNV	Stop gain
SRS-484	LRS-484	TTN	c.72945C>A	p.Tyr24315Ter	SNV	Stop gain
SRS-485	LRS-485	TNNT2	c.388C>T	p.Arg130Cys	SNV	Missense
SRS-486	LRS-486	MYBPC3	c.2157_2158del	p.Cys719Ter	SNV	Stop gain
SRS-487	LRS-487	MYBPC3	c.913_914del	p.Phe305fs	INDEL	Frameshift
SRS-488	LRS-488	TTN	c.2762_2765del	p.Gly921fs	INDEL	Frameshift
SRS-489	LRS-489	MYH7	c.2167C>T	p.Arg723Cys	SNV	Missense
SRS-490	LRS-490	DSC2	c.2686_2687dup	p.Ala897fs	INDEL	Frameshift
SRS-491	LRS-491	SMAD3	c.229G>A	p.Val77Met	SNV	Missense
SRS-492	LRS-492	LAMP2	c.970C>T	p.Pro324Ser	SNV	Missense
SRS-493	LRS-493	TTN	c.26351G>A	p.Trp8784Ter	SNV	Stop gain
SRS-396	LRS-396	SMAD3	c.401-6G>A	-	SNV	Canonical splice variant
SRS-429	LRS-429	TTN	c.96838C>T	p.Gln32280Ter	SNV	Stop gain
SRS-433	LRS-433	FBN1	c.166C>T	p.Pro56Ser	SNV	Missense

SRS-434	LRS-434	TNNT2	c.305G>A	p.Arg102Gln	SNV	Missense
SRS-439	LRS-439	MYBPC3	c.1210C>T	p.Gln404Ter	SNV	Stop gain
SRS-441	LRS-441	FBN1	c.5967T>A	p.Cys1989Ter	SNV	Stop gain
SRS-442	LRS-442	TNNI3	c.400C>T	p.Leu134Phe	SNV	Missense
SRS-443	LRS-443	TTN	c.2207_2219delGATATAAGGA ACG	p.Gly736fs	INDEL	Frameshift
SRS-444	LRS-444	LAMP2	c.970C>T	p.Pro324Ser	SNV	Missense
BRaCA panel						
SRS-033	LRS-033	BRCA2	c.3046G>T	p.Glu1016Ter	SNV	Stop gain
SRS-034	LRS-034	BRCA1	c.547+2T>A	-	SNV	Canonical splice variant
SRS-035	LRS-035	BRCA1	c.5093_5096del	p.Thr1677fs	INDEL	Frameshift
SRS-037	LRS-037	BRCA1	c.65T>C	p.Leu22Ser	SNV	Missense
SRS-038	LRS-038	BRCA2	c.9253dup	p.Thr3085fs	INDEL	Frameshift
SRS-042	LRS-042	BRCA1	c.3700_3704del	p.Val1234fs	INDEL	Frameshift
SRS-043	LRS-043	BRCA2	c.631G>A	p.Val211Ile	SNV	Missense
SRS-044	LRS-044	BRCA2	c.7180A>T	p.Arg2394Ter	SNV	Stop gain
SRS-045	LRS-045	BRCA2	c.7007G>A	p.Arg2336His	SNV	Missense
SRS-046	LRS-046	BRCA2	c.181T>G	p.Cys61Gly	SNV	Missense
SRS-048	LRS-048	BRCA1	c.5093_5096del	p.Thr1677fs	INDEL	Frameshift
SRS-049	LRS-049	BRCA1	c.5093_5096del	p.Thr1677fs	INDEL	Frameshift
SRS-052	LRS-052	BRCA1	c.65T>C	p.Leu22Ser	SNV	Missense
SRS-053	LRS-053	BRCA1	c.3257T>G	p.Leu1086Ter	SNV	Stop gain
SRS-054	LRS-054	BRCA1	c.1088del	p.Asn363fs	INDEL	Frameshift
SRS-055	LRS-055	BRCA1	c.5093_5096del	p.Thr1677fs	INDEL	Frameshift
SRS-056	LRS-056	BRCA1	c.514del	p.Gln172fs	INDEL	Frameshift
SRS-058	LRS-058	BRCA1	c.116G>A	p.Cys39Tyr	SNV	Missense
SRS-059	LRS-059	BRCA1	c.190T>C	p.Cys64Arg	SNV	Missense
SRS-060	LRS-060	BRCA2	c.1310_1313del	p.Lys437fs	INDEL	Frameshift
SRS-061	LRS-061	BRCA1	c.65T>C	p.Gln74Lys	SNV	Missense
SRS-062	LRS-062	BRCA2	c.8878C>T	p.Gln2960Ter	SNV	Stop gain
SRS-063	LRS-063	BRCA1	c.5382dup	p.Asn1774fs	INDEL	Frameshift
SRS-064	LRS-064	BRCA2	c.8878C>T	p.Gln2960Ter	SNV	Stop gain
SRS-081	LRS-081	BRCA1	c.181T>G	p.Cys61Gly	SNV	Missense
SRS-082	LRS-082	BRCA2	c.1216_1219delnsACCG	p.Ala406_Gln407delinsThr Glu	INDEL	DelIns

Table S3: CNVs

SRS-ID	LRS-ID	Gene	DEL/DUP Exons	Detected by ONT
HevaPRO				
SRS-003	LRS-003	ATM	DEL EX 57-59	Detected
SRS-005	LRS-005	BRCA1	DUP EX 12	Detected
SRS-007	LRS-007	PTEN	DEL EX 2-9	Detected
SRS-018	LRS-018	NF1	DEL EX 2-58	Detected
SRS-019	LRS-019	PALB2	DEL EX 12-13	Detected
SRS-021	LRS-021	PMS2	DEL EX 12-15	Detected
SRS-026	LRS-026	RB1	DEL EX 1-27	Detected
SRS-264	LRS-264	NF1	DEL EX 1-58	Detected
SRS-265	LRS-265	MSH6	DUP EX 5-6	Detected
SRS-291	LRS-291	PMS2	DUP EX 1-15	Detected
SRS-301	LRS-301	PALB2	DEL EX 11-13	Detected
SRS-302	LRS-302	PALB2	DEL EX 12-13	Detected
SRS-306	LRS-306	ATM	DUP EX 62-63	Detected
SRS-318	LRS-318	BRCA1	DEL EX 15	Detected
SRS-321	LRS-321	NF1	DEL EX 1-58	Detected
SRS-327	LRS-327	ATM	DEL EX 2-63	Detected
SRS-357	LRS-357	BRCA2	EX 2-14	Detected
SRS-363	LRS-363	BRCA1	DEL EX 1-2	Detected
SRS-366	LRS-366	BRCA1	DUP EX12	Detected
SRS-367	LRS-367	SMAD4	DUP EX2-12	Detected
SRS-378	LRS-378	CHEK2	DEL EX.9-11	Detected
SRS-379	LRS-379	BRIP1	DEL EX7-10	Detected
SRS-454	LRS-454	MSH6	DEL EX4-10	Detected
SRS-457	LRS-457	MRE11	DEL EX2-20	Detected
SRS-473	LRS-473	TP53	DEL EX 2-6	Detected
SRS-494	LRS-494	ATM	DUP EX 50	Detected
CARDIO pro				
SRS-086	LRS-086	LMNA	DEL EX 3-5	Detected
SRS-099	LRS-099	DSP	DEL EX 1-24	Detected
SRS-102	LRS-102	TGFB3	DEL EX 1-7	Detected
SRS-121	LRS-121	ACTA2	DEL EX 2-9	Detected
SRS-132	LRS-132	KCNQ1	DEL EX 9-10	Detected
SRS-133	LRS-133	ACTA2	DEL EX 2-9	Detected
SRS-137	LRS-137	TRDN	DUP EX 1-41	Detected
SRS-138	LRS-138	KCNQ1	DEL EX 9-10	Detected
SRS-154	LRS-154	TGFB2	DEL EX 3	Detected
SRS-170	LRS-170	LMNA	DEL EX 1-12	Detected
SRS-180	LRS-180	COL5A1	DUP EX 1-11	Detected
SRS-181	LRS-181	FBN1	DEL EX 8-66	Detected
SRS-196	LRS-196	NOTCH1	DUP EX 1-34	Detected
SRS-212	LRS-212	MYH11	DUP EX 2-42	Detected
SRS-218	LRS-218	MYH11	DUP EX 2-42	Detected

SRS-229	LRS-229	TGFB2	DEL EX 1-8	Detected
SRS-230	LRS-230	TGFB2	DEL EX 3	Detected
SRS-233	LRS-233	MYH6	DUP EX 3-25	Detected
SRS-236	LRS-236	DMD	DEL EX 45	Detected
SRS-239	LRS-239	ACTA2	DEL EX 1-9	Detected
SRS-242	LRS-242	KCNQ1	DEL EX 7-10	Detected
SRS-256	LRS-256	CBS	DUP EX 3-17	Detected
SRS-385	LRS-385	FLNC	DEL EX 2-24	Detected
SRS-387	LRS-387	DMD	DEL EX 52-53	Detected
SRS-388	LRS-388	KCNQ1	DEL EX 9-10	Detected
SRS-394	LRS-394	FBN1	DEL EX 7-65	Detected
SRS-432	LRS-432	DMD	DEL EX 64-83	Detected
SRS-435	LRS-435	GLA	DEL EX 2-9	Detected
SRS-436	LRS-436	TRDN	DUP EX 1-41	Detected
SRS-437	LRS-437	COL3A1	DEL EX22-24	Detected
SRS-438	LRS-438	FBN1	DEL EX 23	Detected
BRaCA Panel				
SRS-039	LRS-039	BRCA1	DEL EX 21-23	Detected
SRS-040	LRS-040	BRCA1	DEL EX 21-22	Detected
SRS-047	LRS-047	BRCA1	DEL EX 5-10	Detected
SRS-083	LRS-083	BRCA1	DEL EX 1-2	Detected

Table S4: Double and compound LP/P variants

SRS-ID	LRS-ID	Gene	HGVSc or DEL/DUP exon	HGVSp	Variant type
CARDIO pro					
SRS-086	LRS-086	LMNA	DEL EX3-5	-	CNV
		CBS	c.828+1G>A	-	Canonical splice
SRS-088	LRS-088	SMAD3	c.860G>A	p.Arg287Gln	Missense
		SCO2	c.448C>T	p.Gln150Ter	Stop gain
SRS-092	LRS-092	MYBPC3	c.3259A>T	p.Lys1087Ter	Stop gain
		ALMS1	c.2660C>G	p.Ser887Ter	Stop gain
SRS-093	LRS-093	MYH7	c.727C>T	p.Arg243Cys	Missense
		GAA	c.118C>T	p.Arg40Ter	Stop gain
SRS-097	LRS-097	COL3A1	c.2105G>A	p.Gly702Asp	Missense
		DSG2	c.523+1G>A	-	Canonical splice
SRS-106	LRS-106	MYBPC3	c.1624G>C	p.Glu542Gln	Missense
		CBS	DUP EX3-17	-	CNV
SRS-108	LRS-108	LAMA2	c.4936G>T	p.Glu1646Ter	Stop gain
		COX15	c.784C>T	p.Arg262Ter	Stop gain
SRS-109	LRS-109	FBN1	c.3337+1G>A	-	Canonical splice
		COL3A1	c.1966C>A	p.Pro656Thr	Missense
SRS-112	LRS-112	MYH7	c.2572C>T	p.Arg858Cys	Missense
		MYBPC3	c.237C>G	p.Tyr79Ter	Stop gain
		DSP	c.8007_8008del	p.Ser2670fs	Frameshift
SRS-114	LRS-114	LMNA	c.568C>T	p.Arg190Trp	Missense
		FLNC	c.6799G>A	p.Val2267Ile	Missense
SRS-118	LRS-118	TTN	c.104069del	p.Gly34690fs	Frameshift
		TGFB3	c.1130G>A	p.Cys377Tyr	Missense
SRS-120	LRS-120	RYR1	c.14903C>T	p.Pro4968Leu	Missense
		MYH7	c.3791A>G	p.Glu1264Gly	Missense
SRS-123	LRS-123	ACTA2	c.169G>A	p.Gly57Ser	Missense
		TRIM63	c.739C>T	p.Gln247Ter	Stop gain
SRS-232	LRS-232	MYBPC3	c.2429G>A	p.Arg810His	Missense
		MYBPC3	c.3331-26T>G	-	Non canonical splice
SRS-233	LRS-233	MYH6	DUP EX 3-25	-	CNV
		MYH7	DUP EX 28-40	-	CNV
SRS-240	LRS-240	FBN1	c.6662G>A	p.Cys2221Tyr	Missense
		COL3A1	c.4090G>A	p.Ala1364Thr	Missense
SRS-241	LRS-241	MYH7	c.2287G>A	p.Val763Met	Missense
		MYBPC3	c.1224-80G>A	-	Intronic
SRS-244	LRS-244	MYBPC3	c.98_99delCA	p.Thr33fs	Frameshift
		MYBPC3	c.1809T>G	p.Ile603Met	Missense
SRS-245	LRS-245	MYH6	c.2326_2329dup	p.Glu777fs	Frameshift
		MYH6	c.1669C>A	p.His557Asn	Missense
		LDLR	c.539A>G	p.Asp180Gly	Missense
SRS-247	LRS-247	SCN5A	c.733C>A	p.Gln245Lys	Missense
		PSEN1	DUP EX3	-	CNV
SRS-132	LRS-132	RYR2	c.1976T>G	p.Ile659Ser	Missense
		KCNQ1	DEL 9-10	-	CNV
SRS-138	LRS-138	KCNQ1	DEL 9-10	-	CNV
		RYR2	c.1976T>G	p.Ile659Ser	Missense
SRS-140	LRS-140	FBN1	c.4337-1G>A	-	Canonical splice
		MYH7	c.5287G>A	p.Ala1763Thr	Missense
SRS-148	LRS-148	LMNA	c.1634G>A	p.Arg545His	Missense

		MYBPC3	c.506-2A>G	-	Canonical splice
SRS-158	LRS-158	LMNA	c.1510G>T	p.Ala504Ser	Missense
		TNNT2	c.586C>T	p.Arg196Trp	Missense
SRS-161	LRS-161	MYBPC3	c.1828G>C	p.Asp610His	Missense
		DSP	c.6137T>G	p.Val2046Gly	Missense
SRS-162	LRS-162	MYBPC3	c.772G>A	p.Glu258Lys	Missense
		ALPK3	c.1307del	p.Gly436fs	Frameshift
SRS-439	SRS-439	MYBPC3	c.1210C>T	p.Gln404Ter	Stop gain
		MYH6	c.3913del	p.Arg1305fs	Frameshift
SRS-441	SRS-441	FBN1	c.5967T>A	p.Cys1989Ter	Stop gain
		SLC2A10	c.1330C>T	p.Arg444Ter	Stop gain
SRS-382	LRS-382	MYH7	c.2167C>A	p.Arg723Ser	Missense
		MYBPC3	c.2157_2158del	p.Cys719fs	Frameshift
SRS-384	LRS-384	LMNA	c.481G>A	p.Glu161Lys	Missense
		KCNQ1	c.568C>T	p.Arg190Trp	Missense
SRS-389	LRS-389	DES	c.1048C>T	p.Arg350Trp	Missense
		LMNA	c.1774G>A	p.Gly592Arg	Missense
SRS-390	LRS-390	TTN	c.72945C>A	p.Tyr24315Ter	Stop gain
		LMNA	c.1324G>A	p.Val442Met	Missense
SRS-395	LRS-395	TNNI3	c.607C>T	p.Arg203Cys	Missense
		SCN5A	c.4501C>G	p.Leu1501Val	Missense
SRS-383	LRS-383	TTN	c.42527_42565delinsTCCC	p.Lys14176fs	Frameshift
		TTN	c.39749_39766del	p.Ile13250_Glu13255del	Inframe deletion
SRS-490	LRS-490	DSC2	c.2686_2687dup	p.Ala897fs	Frameshift
		TTN	c.55332del	p.Ile18445fs	Frameshift
HEVA pro					
SRS-004	LRS-004	PALB2	c.2996+1G>T	-	Canonical splice
		BARD1	c.1921C>T	p.Arg641Ter	Stop gain
SRS-020	LRS-020	PALB2	c.661_662delinsTA	p.Val221Ter	Stop gain
		CHEK2	c.1388G>A	p.Cys463Tyr	Missense
SRS-006	LRS-006	BRIP1	c.3525dupT	p.Ile1176fs	Frameshift
		BRCA2	c.5851_5854del	p.Ser1951fs	Frameshift
SRS-015	LRS-015	RAD51C	c.935G>A	p.Arg312Gln	Missense
		MSH6	c.2677_2678del	p.Leu893fs	Frameshift
SRS-285	LRS-285	BRCA1	c.190T>C	p.Cys64Arg	Missense
		BRCA2	c.7180A>T	p.Arg2394Ter	Stop gain
SRS-293	LRS-293	BRCA1	c.514del	p.Gln172fs	Frameshift
		CHEK2	c.599T>C	p.Ile200Thr	Missense
SRS-287	LRS-287	MSH2	c.1022_1029del	p.Leu341fs	Frameshift
		BARD1	DEL EX 3	-	CNV
SRS-341	LRS-341	ATM	c.4906C>T	p.Gln1636Ter	Stop gain
		BRIP1	c.3086del	p.Ser1029fs	Frameshift
SRS-361	LRS-361	BRCA2	c.9218A>C	p.Asp3073Ala	Missense
		WRN	c.3913C>T	p.Arg1305Ter	Stop gain
SRS-375	LRS-375	PALB2	c.1317delG	p.Phe440fs	Frameshift
		NF2	c.240+1G>T	-	Canonical splice
SRS-382	LRS-382	MYH7	c.2167C>A	p.Arg723Ser	Missense
		MYBPC3	c.2157_2158del	p.Cys719fs	Frameshift
SRS-384	LRS-384	LMNA	c.481G>A	p.Glu161Lys	Missense
		KCNQ1	c.568C>T	p.Arg190Trp	Missense
SRS-389	LRS-389	DES	c.1048C>T	p.Arg350Trp	Missense
		LMNA	c.1774G>A	p.Gly592Arg	Missense
SRS-390	LRS-390	TTN	c.72945C>A	p.Tyr24315Ter	Stop gain

		LMNA	c.1324G>A	p.Val442Met	Missense
SRS-395	LRS-395	TNNI3	c.607C>T	p.Arg203Cys	Missense
		SCN5A	c.4501C>G	p.Leu1501Val	Missense
SRS-383	LRS-383	TTN	c.42527_42565delinsTCCC	p.Lys14176fs	Frameshift
		TTN	c.39749_39766del	p.Ile13250_Glu13255del	Inframe deletion
SRS-027	LRS-027	MUTYH	c.1103G>A	p.Gly368Asp	Missense
		MUTYH	c.228C>A	p.Tyr76Ter	Stop gain
SRS-140	LRS-140	FBN1	c.4337-1G>A	-	Canonical splice
		MYH7	c.5287G>A	p.Ala1763Thr	Missense
SRS-412	LRS-412	RAD51C	c.109G>T	p.Glu37Ter	Stop gain
		BARD1	c.513dupA	p.Asp172fs	Frameshift
SRS-452	SRS-452	MUTYH	c.536A>G	p.Tyr179Cys	Missense
		ATM	c.2921+1G>A	.	Canonical splice

Table S5: List of deep intronic variants reported as LP/P in ClinVar within genes included in analyzed panels as detectable with SRS and ONT.

**Gene**	**Variant**	**ClinVar classification**	**SRS ≥ 1x**	**SRS ≥ 10X**	**ONT ≥ 1X**	**ONT ≥ 10X**
			**HEVA pro**			
APC	c.1408+729A>G	Pathogenic	No	No	**Yes**	**Yes**
APC	c.1408+731C>T	Likely pathogenic	No	No	**Yes**	**Yes**
APC	c.1408+743_1408+745delinsACG	Likely pathogenic	No	No	**Yes**	**Yes**
APC	c.532-934A>T	Likely pathogenic	No	No	**Yes**	**Yes**
APC	c.933+829A>G	Likely pathogenic	No	No	**Yes**	**Yes**
APC	c.166-28467del	Pathogenic	No	No	**Yes**	**Yes**
ATM	c.1236-404C>T	Likely pathogenic	No	No	**Yes**	**Yes**
ATM	c.1803-270T>G	Pathogenic	No	No	**Yes**	**Yes**
ATM	c.2125-910T>G	Likely pathogenic	No	No	**Yes**	**Yes**
ATM	c.3994-161G>A	Likely pathogenic	No	No	**Yes**	**Yes**
ATM	c.496+286A>G	Likely pathogenic	No	No	**Yes**	**Yes**
ATM	c.496+334A>G	Likely pathogenic	No	No	**Yes**	**Yes**
ATM	c.496+338G>A	Likely pathogenic	No	No	**Yes**	**Yes**
ATM	c.497-1142dup	Likely pathogenic	No	No	**Yes**	No
ATM	c.5178-296A>G	Likely pathogenic	No	No	**Yes**	**Yes**
BRCA1	c.5277+833_5277+1671delins141	Pathogenic	No	No	**Yes**	No
BRCA2	c.7618-187G>T	Likely pathogenic	No	No	**Yes**	**Yes**
CDKN2A	c.458-521G>T	Pathogenic	**Yes**	**Yes**	**Yes**	**Yes**
CHEK2	c.444+119del	Pathogenic	**Yes**	**Yes**	**Yes**	**Yes**
CHEK2	c.1009-1264C>G	Pathogenic	No	No	**Yes**	No
MLH1	c.116+96C>T	Likely pathogenic	**Yes**	**Yes**	**Yes**	**Yes**
MLH1	c.1732-264A>T	Pathogenic	No	No	**Yes**	**Yes**
MRE11	c.1226-912C>T	Likely pathogenic	No	No	**Yes**	**Yes**
MSH2	c.1387-2012C>T	Likely pathogenic	No	No	No	No
MSH2	c.212-478T>G	Pathogenic	No	No	**Yes**	**Yes**
MSH2	c.2458+976A>G	Likely pathogenic	No	No	**Yes**	**Yes**
NF1	c.1260+1604A>G	Pathogenic	No	No	**Yes**	No
NF1	c.1527+675C>T	Likely pathogenic	No	No	**Yes**	**Yes**
NF1	c.1846-569A>C	Pathogenic	No	No	**Yes**	**Yes**
NF1	c.288+1137C>T	Pathogenic	No	No	**Yes**	No
NF1	c.288+2025T>G	Pathogenic	No	No	No	No
NF1	c.288+545A>G	Likely pathogenic	No	No	**Yes**	**Yes**
NF1	c.4110+945A>G	Pathogenic	No	No	**Yes**	**Yes**
NF1	c.4577+357T>G	Pathogenic	No	No	**Yes**	**Yes**
NF1	c.5813-177A>C	Likely pathogenic	**Yes**	**Yes**	**Yes**	**Yes**
NF1	c.5813-184_5813-178dup	Pathogenic	**Yes**	**Yes**	**Yes**	**Yes**
NF1	c.60+18227_60+18228ins32	Pathogenic	No	No	**Yes**	No
PALB2	c.3113+7271del	Pathogenic	**Yes**	**Yes**	**Yes**	**Yes**
PTEN	c.209+2047A>G	Likely pathogenic	No	No	No	No
RAD51D	c.263+1617C>T	Pathogenic	**Yes**	**Yes**	**Yes**	**Yes**

RB1	c.1695+30914_1695+30917dup	Pathogenic	No	No	No	No
RB1	c.2490-1398A>G	Pathogenic	No	No	**Yes**	No
SMARCB1	c.500+883T>G	Pathogenic	No	No	**Yes**	**Yes**
SMARCB1	c.500+887G>A	Pathogenic	No	No	**Yes**	**Yes**
**CARDIO pro**						
COL5A1	c.5136+68_5136+73delinsT	Likely pathogenic	**Yes**	**Yes**	**Yes**	**Yes**
FBN1	c.1469-244T>G	Pathogenic	No	No	**Yes**	**Yes**
FBN1	c.3965-1081A>G	Likely pathogenic	No	No	**Yes**	**Yes**
FBN1	c.5422+452C>T	Pathogenic	No	No	**Yes**	**Yes**
FBN1	c.5671+773C>G	Pathogenic	No	No	**Yes**	**Yes**
FBN1	c.6617-147G>A	Pathogenic	**Yes**	**Yes**	**Yes**	**Yes**
FBN1	c.6872-955C>G	Likely pathogenic	No	No	**Yes**	**Yes**
GLA	c.640-394C>T	Likely pathogenic	No	No	**Yes**	**Yes**
GLA	c.640-859C>T	Likely pathogenic	**Yes**	**Yes**	**Yes**	**Yes**
KCNQ1	c.1514+37364_1514+38744del	Pathogenic	No	No	No	No
MYBPC3	c.1090+453C>T	Likely pathogenic	No	No	**Yes**	**Yes**
MYBPC3	c.1927+337G>T	Likely pathogenic	No	No	**Yes**	**Yes**
MYBPC3	c.1927+600C>T	Pathogenic	No	No	**Yes**	**Yes**
MYBPC3	c.2905+445_2905+448del	Pathogenic	No	No	**Yes**	**Yes**
LAMA2	c.5071+3104del	Pathogenic	No	No	No	No
ELN	c.1747+100G>C	Likely pathogenic	**Yes**	**Yes**	**Yes**	**Yes**
ELN	c.1747+100del	Likely pathogenic	**Yes**	**Yes**	**Yes**	**Yes**
LDLR	c.2141-218G>A	Likely pathogenic	No	No	**Yes**	**Yes**
RYR1	c.14647-1449A>G	Pathogenic	No	No	No	No
RYR1	c.3178+587A>G	Likely pathogenic	No	No	**Yes**	**Yes**
PKP2	c.1379-1992C>T	Pathogenic	**Yes**	**Yes**	**Yes**	No
DSP	c.3582+1242dup	Likely pathogenic	**Yes**	**Yes**	**Yes**	**Yes**
SCN1B	c.448+349del	Likely pathogenic	**Yes**	**Yes**	**Yes**	**Yes**
CRELD1	c.1049-401C>A	Pathogenic	No	No	**Yes**	**Yes**
FKTN	c.165+1427A>G	Pathogenic	**Yes**	**Yes**	**Yes**	**Yes**
FLNA	c.2280+389T>A	Pathogenic	No	No	**Yes**	**Yes**
ANK2	c.85-56996T>C	Likely pathogenic	**Yes**	**Yes**	**Yes**	**Yes**
ZIC3	c.1224+3286A>G	Pathogenic	No	No	No	No
DES	c.1289-741G>A	Pathogenic	No	No	**Yes**	**Yes**
DMD	c.1812+601A>G	Likely pathogenic	No	No	**Yes**	**Yes**
DMD	c.2292+1024G>T	Likely pathogenic	No	No	**Yes**	**Yes**
DMD	c.265-463A>G	Pathogenic	No	No	**Yes**	**Yes**
DMD	c.3603+2053G>C	Likely pathogenic	No	No	No	No
DMD	c.3603+820G>T	Pathogenic	No	No	**Yes**	**Yes**
DMD	c.4072-267del	Pathogenic	**Yes**	**Yes**	**Yes**	**Yes**
DMD	c.5155-719_5155-31del	Likely pathogenic	**Yes**	**Yes**	**Yes**	**Yes**
DMD	c.6290+3076A>G	Pathogenic	No	No	No	No
DMD	c.6291-13537A>G	Likely pathogenic	No	No	**Yes**	No
DMD	c.6614+3310G>T	Likely pathogenic	No	No	No	No
DMD	c.8217+18052A>G	Likely pathogenic	No	No	No	No

DMD	c.8217+32103G>T	Likely pathogenic	No	No	No	No
DMD	c.832-186T>G	Pathogenic	**Yes**	**Yes**	**Yes**	**Yes**
DMD	c.9163+2510G>A	Likely pathogenic	No	No	No	No
DMD	c.9974+175T>A	Pathogenic	**Yes**	No	**Yes**	**Yes**
NKX2-5	c.335-204del	Likely pathogenic	**Yes**	**Yes**	**Yes**	**Yes**
MYO6	c.2417-1758T>G	Pathogenic	No	No	**Yes**	No
TRDN	c.484+1189G>A	Likely pathogenic	**Yes**	**Yes**	**Yes**	**Yes**
LDLRAP1	c.748-608G>A	Likely pathogenic	No	No	**Yes**	**Yes**
PRKAG2	c.466+45171G>T	Likely pathogenic	**Yes**	**Yes**	**Yes**	**Yes**
TBX5	c.664-342G>T	Likely pathogenic	No	No	**Yes**	**Yes**
